# Profile of Chromosomal Alterations, Chromosomal Instability and Clonal Heterogeneity in Colombian Farmers Exposed to Pesticides

**DOI:** 10.3389/fgene.2022.820209

**Published:** 2022-02-24

**Authors:** María Paula Meléndez-Flórez, Duvan Sebastián Valbuena, Sebastián Cepeda, Nelson Rangel, Maribel Forero-Castro, María Martínez-Agüero, Milena Rondón-Lagos

**Affiliations:** ^1^ School of Biological Sciences, Universidad Pedagógica y Tecnológica de Colombia, Tunja, Colombia; ^2^ Departamento de Nutrición y Bioquímica, Facultad de Ciencias, Pontificia Universidad Javeriana, Bogotá, Colombia; ^3^ Centro de Investigaciones en Microbiología y Biotecnología-UR (CIMBIUR), Facultad de Ciencias Naturales, Universidad del Rosario, Bogotá, Colombia

**Keywords:** pesticides, chromosomal alterations, chromosomal instability, clonal heterogeneity, occupational exposure

## Abstract

Pesticides are a group of environmental pollutants widely used in agriculture to protect crops, and their indiscriminate use has led to a growing public awareness about the health hazards associated with exposure to these substances. In fact, exposure to pesticides has been associated with an increased risk of developing diseases, including cancer. In a study previously published by us, we observed the induction of specific chromosomal alterations and, in general, the deleterious effect of pesticides on the chromosomes of five individuals exposed to pesticides. Considering the importance of our previous findings and their implications in the identification of cytogenetic biomarkers for the monitoring of exposed populations, we decided to conduct a new study with a greater number of individuals exposed to pesticides. Considering the above, the aim of this study was to evaluate the type and frequency of chromosomal alterations, chromosomal variants, the level of chromosomal instability and the clonal heterogeneity in a group of thirty-four farmers occupationally exposed to pesticides in the town of Simijacá, Colombia, and in a control group of thirty-four unexposed individuals, by using Banding Cytogenetics and Molecular Cytogenetics (Fluorescence *in situ* hybridization). Our results showed that farmers exposed to pesticides had significantly increased frequencies of chromosomal alterations, chromosomal variants, chromosomal instability and clonal heterogeneity when compared with controls. Our results confirm the results previously reported by us, and indicate that occupational exposure to pesticides induces not only chromosomal instability but also clonal heterogeneity in the somatic cells of people exposed to pesticides. This study constitutes, to our knowledge, the first study that reports clonal heterogeneity associated with occupational exposure to pesticides. Chromosomal instability and clonal heterogeneity, in addition to reflecting the instability of the system, could predispose cells to acquire additional instability and, therefore, to an increased risk of developing diseases.

## Introduction

Pesticides are a group of environmental pollutants widely used in agriculture to protect crops, so their indiscriminate use has led to a growing public awareness about the health hazards associated with exposure to these substances. Additionally, given that in Colombia one of the most important economic activities is agriculture, occupational exposure to these pesticides constitutes a risk due to their detrimental effect on human health. Currently, there are more than 1000 chemicals, which are classified as pesticides, some of them considered as potential genotoxic agents. Although the World Health Organization (WHO), groups pesticides according to their potential health risks (FAO and WHO, 2021), several of the classified as extremely toxic, are still used in our country, Colombia, including herbicide, fungicide and insecticide (mancozeb, glyphosate, malathion) (Idrovo, 2000). Pesticide exposure (absorption *via* dermal and/or respiratory routes) is now known to be associated with genotoxicity, oxidative stress, genetic damage and induction of chromosomal alterations, as well as reproductive disorders, neurodegenerative and cardiovascular diseases, and even with an increased carcinogenic risk (Cocco et al., 2013; Nicolopoulou-Stamati et al., 2016; Polito et al., 2016), especially for hematopoietic bone marrow cancers including myelodysplastic syndrome (MDS), leukemia acute myeloid (AML) and multiple myeloma (Tomiazzi et al., 2018). In fact, genetic damage constitutes an important event in the development of carcinogenesis, also correlated with the induction of genomic instability. Chromosomal damage related to pesticide exposure, has been identified in several populations, and while some researchers have reported significant differences in the frequency of chromosomal alterations (CAs) in exposed individuals compared to unexposed controls (Dulout et al., 1985; Carbonell et al., 1990; De Ferrari et al., 1991; Rupa et al., 1991; Balaji and Sasikala, 1993; Brega et al., 1998), others have not observed any association (Gomez-Arroyo et al., 2000). However, in these studies, the evaluation of chromosomal damage has been limited to the identification of chromosome gaps, breaks, sister chromatid exchange (Gomez-Arroyo et al., 2000) and micronuclei (MN), among others, so information on the type and frequency of specific CAs and chromosomal variants (CVs), as well as the level of chromosomal instability (CIN) and clonal heterogeneity (CH) induced by exposure to pesticides is scarce. In fact, one of the few studies available that indicate the type and frequency of specific chromosomal alterations induced by exposure to pesticides was reported by us, in a small group of exposed (five exposed) (Cepeda et al., 2020). Considering the importance of our previous findings (Cepeda et al., 2020) and their implications both, in the identification of cytogenetic biomarkers for the monitoring of exposed populations, and in the possibilities of their future application in early diagnostic tests, we decided to conduct a new study with a greater number of individuals exposed to pesticides. Considering the above, the aim of the present study was to evaluate the genotoxic damage (CAs, CVs, CIN and CH), in a group of thirty-four (34) farmers occupationally exposed to pesticides in the town of Simijacá, Colombia, and in a control group of thirty-four (34) unexposed individuals, by using GTG Banding and Fluorescence *in situ* hybridization (FISH). The results obtained from the analysis of a large number of metaphases, allowed to identify the type and frequency of CAs and CVs, as well the level of CIN and CH, not previously reported in farmers exposed to pesticides. Our study shows the deleterious effect of pesticides on the chromosomes of occupationally exposed individuals.

## Materials and Methods

### Study Population

A total of 68 individuals were part of this study: thirty-four (34) individuals from the town of Simijacá, Colombia who were farmers routinely “exposed” to pesticides (exposed group) and thirty-four (34) individuals without indication of previous occupational exposure to pesticides (unexposed group). The exposed group consisted of men and women between 23 and 70 years old, involved in pesticide spray/handling and who had been exposed to pesticides through work for at least 3 months. The farmers’ route of exposure to pesticides was mainly dermal and/or respiratory (Table 1 and Supplementary Table S1). Minor routes of exposure to pesticides, including unintentional (accidental) oral exposure, ocular/ear exposure, and/or parenteral exposure (intramuscular, subcutaneous, or intravenous), were not reported by the exposed group. The unexposed group consisted of healthy men and women, without indication of previous occupational exposure to pesticides. The unexposed group had a similar age range (between 23 and 70 years old), sex distribution and life style habits as the exposed group (Table 1 and Supplementary Table S1). Each subject was also required to complete a routine questionnaire to record possible confounding factors such as diseases, age, smoking and drinking habits, time of exposure to pesticides, pesticide exposure frequency, type of pesticide mixture, the dose of pesticides (expressed in kilograms/hectare) used by each exposed individual, as well as the number of hectares sprayed per day by each of them (Table 1 and Supplementary Table S1). Participants suffering from cancer or had received radiotherapy, chemotherapy, or other prolonged medical treatment, were excluded from the study. Data from the exposed individuals were compared with those of the unexposed individuals.

**TABLE 1:** General characteristics of the groups studied.

	Exposed	Unexposed
Number	34	34
Age (mean ± SD)	46.64 ± 12.13	47.11 ± 11.24
Sex (n)		
Male	20	20
Female	14	14
Exposure months (mean ± SD)	133.2 ± 126.6	0
Smoking status (n)		
Smokers	4	4
Non-smokers	30	30
Drinking status (*n*)		
Drinkers	25	17
Non-drinkers	9	20

SD, standard deviation.

### Blood Sampling

Five milliliters of peripheral blood, from exposed and unexposed individuals, were collected into heparinized tubes by venous puncture. The written informed consent of each subject participating in the study was obtained before the blood samples were taken.

### Cytogenetic Studies and GTG Banded Karyotyping

The metaphases and interphase nuclei of the cultured peripheral blood lymphocytes were obtained using standard protocols. Briefly, lymphocyte cultures were performed by adding 1 ml of whole blood, in 5 ml of RPMI-1640 medium (Sigma, St. Louis, MO, United States), supplemented with 10% fetal bovine serum (FBS) (Sigma) and 100 μl of phytohemagglutinin-M (Gibco, Life Technologies, Nebraska, United States). The cultures were incubated at 37°C for 72 h in a 5% CO_2_ atmosphere. All cultures of each individual, exposed and unexposed, were performed in duplicate. After 72 h, a solution of N-deacetyl-N-methyl colchicine (0.0001 g/ml final concentration) (Sigma) was added to the cultures for 25 min. After this time, the cells were treated with hypotonic solution (0.075 M KCl) for and fixed with carnoy fixative (3:1 methanol: acetic acid). Thus obtained, the chromosomal preparations were spread on glass slides and banded with GTG banding using trypsin (0.25%) (Gibco) and Giemsa (Sigma).

### Cytogenetic Analysis

The identification of CVs and CAs (numerical and structural chromosomal alterations), by using GTG banded karyotyping was performed on a total of 2554 metaphases. Metaphase spreads were analyzed using an Olympus microscope and processed using the cytogenetic software Cytovision System 7.4 (Leica Biosystems Richmond, VA, United States). CVs [variation in length of heterochromatic segments on the long arms of chromosomes 1 (1qh+), 9 (9qh+) and 16 (16qh+)], fragilities (fra), inversion of chromosome 9 [inv(9)], chromosomal breaks (chrb) and chromatid breaks (chrb), and CAs including structural (SCAs) and numerical chromosomal alterations (NCAs) were evaluated. All CVs and CAs were described according to the International System for Human Cytogenomic Nomenclature (ISCN) 2020 (McGowan-Jordan et al., 2020).

### Molecular Cytogenetics Studies (FISH)

FISH was used to evaluate CIN and CH on chromosomal spreads (metaphases and interphase nuclei) previously obtained. For the above, six (6) centromeric probes (CEP) labeled with different fluorochromes were used, for chromosomes 2 and 3 (orange fluorochrome), 8 and 17 (blue fluorochrome) and, 11 and 15 (green fluorochrome) (all from Cytocell, Cambridge). Tricolor FISH was performed on the chromosome preparations for chromosomes 2, 8, and 11, and for chromosomes 3, 15, and 17. Briefly, the chromosomal spreads were dehydrated in ethanol series, and after adding the probe mixture, they were denaturated at 75°C for 2 min and hybridized overnight at 37°C, using the Top Brite system (Resnova, Italy). After this time, the chromosome extensions were washed, dehydrated and stained with 4′, 6-diamidino-2-phenylindole (Cytocell). Finally, ten randomly selected areas of the chromosomal spreads from each exposed and unexposed individual, were acquired using an Olympus microscope and processed using the cytogenetic software Cytovision System 7.4. CIN was evaluated in a minimum of 100 intact and non-overlapping nuclei/metaphases for each chromosome. Although it has been suggested that the use of probes for only two chromosomes is sufficient to identify diploid aneuploid tumors (Fiegl et al., 2000; Takami et al., 2001), we decided to use 6 probes because the use of more than two probes allows the identification of clonal populations with greater certainty (Farabegoli et al., 2001). The CIN rate for each exposed and unexposed individual was defined first by calculating, for each of the six chromosomes separately, the percentage of nuclei with a CEP signal number different to the modal number (most frequent number of chromosomes in a cell population), and then calculating the mean CIN percentage of all six chromosomes analyzed (Lengauer et al., 1997; Munro et al., 2012). According to the level of CIN, each exposed and unexposed individual was classified as having low CIN (CIN < 25%) or high CIN (CIN ≥ 25%) (Kawauchi et al., 2010; Talamo et al., 2010). The CIN levels observed in each of exposed individuals were determined in comparison with the control group (unexposed). In order to evaluate the CH (presence of cell populations with different levels of aneuploidy in the same person), in each exposed and unexposed individual, we calculated the Shannon Diversity Index (SDI) and the true diversity index (TD) for chromosomes 2, 3, 8, 11, 15, and 17. SDI and TD integrates both the number and abundance of cell clones within each cell according to published methods (Jost, 2006; Maley et al., 2006; Roylance et al., 2011).

### Data Analysis

With the aim of comparing the GTG-banding cytogenetic data with parametric and non-parametric distribution, Fisher’s exact test, Student’s t-test and Wilcoxon test were performed. Normality of the data was evaluated by the Shapiro Wilk test. Data from the exposed individuals were compared with those of the unexposed individuals. Student’s t-test and Wilcoxon test were performed to compare CIN, SDI, and TD data with parametric and nonparametric distribution, respectively. To compare CIN, SDI, and TD between the chromosomes used in this study, the Kruskal–Wallis test was used for data with nonparametric distribution. Normality and homoscedasticity of the data were assessed by Shapiro Wilk’s test and Bartlett’s test, respectively. In order to establish, in each of the exposed and unexposed groups, the existence of associations between the levels of CIN and CH with variables such as age, sex, and time of exposure to pesticides (only in exposed), we perform multivariate analysis using the Pearson correlation coefficient. Data from exposed individuals were compared with those from unexposed individuals. All statistical analyses were carried out using the R Studio version 4.0.2 and *p* values < 0.05 were considered as statistically significant (*p ≤ 0.05, **p ≤ 0.01 and ***p ≤ 0.001). CIN, SDI and TD are expressed as means ± SD.

## Results

### Characteristics of Study Groups

General and detailed characteristics of the groups studied (exposed and unexposed) are presented in Table 1 and Supplementary Table S1, respectively. For the exposed group, the mean time of exposure to pesticides was 133.2 months, the mean age was 46.64 years (Table 1), and the pesticide exposure frequency was mainly once a week (Supplementary Table S1). The dose of pesticides (expressed in kilograms/hectare) used by each exposed individual, as well as the number of hectares sprayed per day by each of them, are also indicated in Supplementary Table S1. A low prevalence of alcohol consumption and cigarette smoking was reported in both groups, exposed and unexposed. The results are expressed as the mean ± standard deviation (SD) (Table 1 and Supplementary Table S1). Pesticides mixtures to which farmers were mainly exposed included: fungicides (Antracol, Cymoxanil, Cymozeb, Dithane, Fitoraz, Forum, Mancozeb, Propineb), insecticides (Arrivo, Astuto, Carbosulfan, Carbofuran, Cayenne, Chlorpyrifos, Confidor, Cypermethrin, Curacron, Decis, Eltra, Engeo, Fulminator, Furadan, Imidacloprid, Karate, Lambda-cyhalothrin, Lannate, Lorsban, Match, Methyl parathion, Perban, Profenofos, Tiguvon), and herbicides (Paraquat, Cerillo) (Supplementary Table S1).

### GTG Banding Cytogenetic Results

**FIGURE 1:**
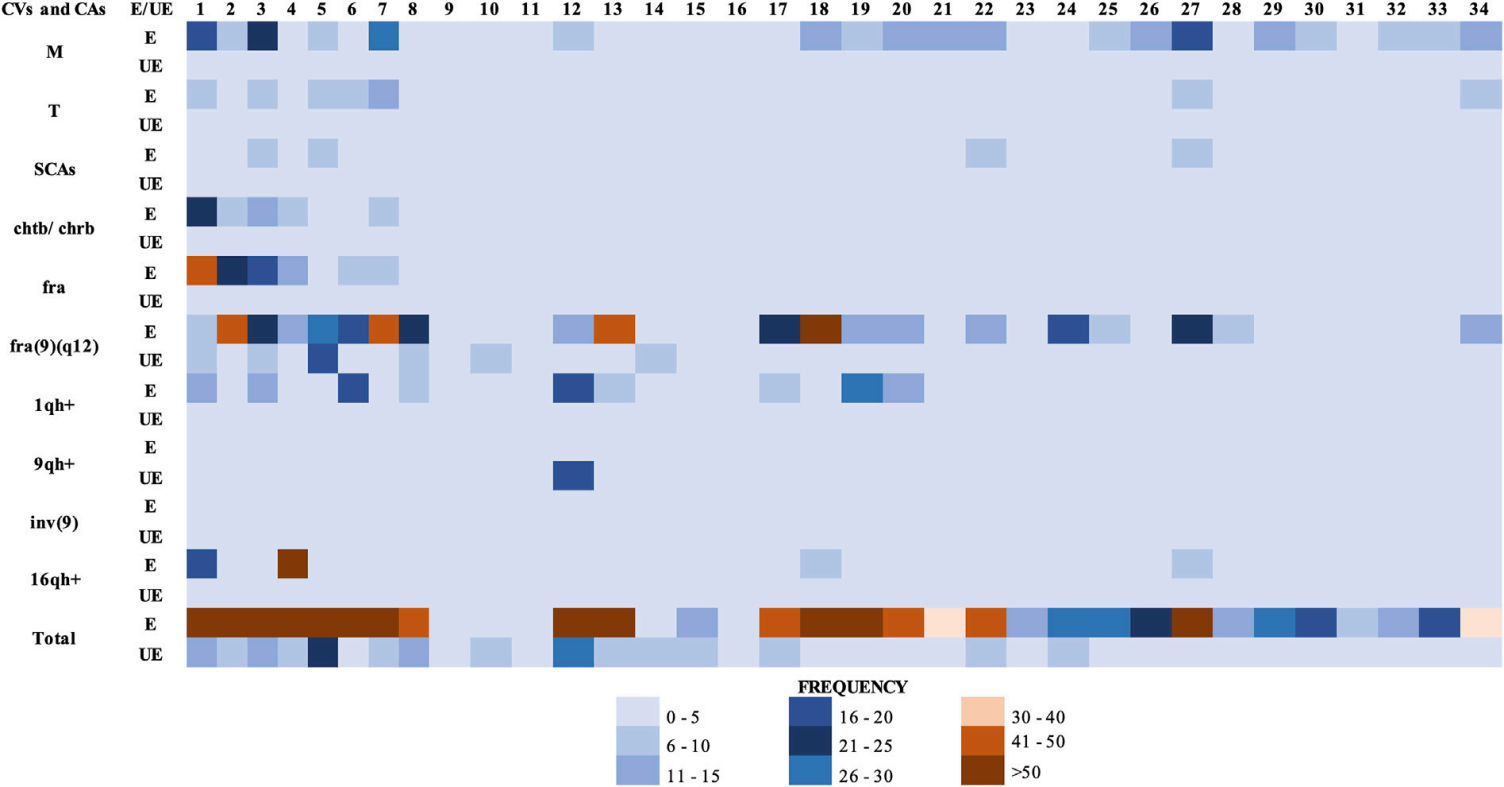
Total chromosomal variants (CVs) and chromosomal alterations (CAs) observed in the groups studied. (E) Exposed group. (UE) Unexposed group. Each column in the figure, represents a participant in the study (34 columns in total). Abbreviations: M, monosomies; T, trisomies; SCAs, structural chromosomal alterations; chtb, chromatidic break; chrb, chromosomic break; fra, fragilities; fra(9)(q12), fragility in the long arm of chromosome 9, region 1 and band 2; 1qh+, heterochromatin increased on long arm of chromosome 1; 9qh+, heterochromatin increased on long arm of chromosome 9; inv(9), inversion of chromosome 9; 16qh+, heterochromatin increased on long arm of chromosome 16.

According to the International recommendations for the analysis of constitutional studies (CCMG-CCGM National Office, 2021; Ozkan and Marcelo, 2021), a minimum of between 10 and 20 metaphases must be analyzed for cytogenetic analysis. If in these 10 or 20 metaphases no numerical or structural alterations are observed, it is not necessary to analyze additional metaphases. If, on the contrary, numerical and/or structural alterations are observed (conditions where mosaicism is a significant possibility), examination of additional metaphases is required (minimum of 25–50 metaphases). Considering the above, we analyzed a minimum of 19 metaphases, from individuals of both groups (exposed and unexposed), in those cases in which no numerical or structural alterations were observed, and we extended the cytogenetic analysis to a maximum of 95 metaphases in the cases in which this type of alterations was observed. The difference in the number of metaphases analyzed is also due to the variation in the mitotic index in each individual included in the study. A total of 2554 metaphases were analyzed. GTG banding cytogenetic analysis for both, exposed and unexposed groups, demonstrated a modal diploid number (2n). Significantly high frequencies for CVs, fragilities, chrb, chrb, structural (SCAs) and numerical chromosomal alterations (NCAs), were found in the exposed group compared with those observed in the unexposed group (1471 and 209, respectively) (*p* ≤ 0.0027**; unpaired Mann-Whitney test) (Figure 1).

Specifically, in the exposed group were observed: 384 numerical alterations in 32 (94.1%) individuals; 88 structural alterations in 27 (79.4%) individuals; 625 fragilities in 32 (94.1%) individuals; 107 chromatid and/or chromosomal breaks in 25 (73.5%) individuals, and 267 chromosomal heteromorphisms in 20 (58.8%) individuals (Table 2). While in the unexposed group, were observed 43 numerical alterations in 15 (44.1%) individuals; 13 structural alterations in 9 (26.4%) individuals; 97 fragilities in 17 (50%) individuals; 26 chromatid and/or chromosomal breaks in 14 (41.1%) individuals, and 30 chromosomal heteromorphisms in 4 (11.8%) individuals (Figure 1 and Table 2). The comparison in the frequency of CVs and CAs between the exposed and unexposed groups showed statistically significant differences (*p* ≤ 0.01**; Fisher’s exact test) in most cases.

**TABLE 2:** Frequencies and percentages of chromosomal variants (CVs) and chromosomal alterations (CAs) identified in the exposed and unexposed groups.

CVs and CAs	Number of individuals		
	Exposed n (%)	Unexposed n (%)	*p*
Monosomies	32 (94.1)	13 (38.2)	<0.0001**
Trisomies	26 (76.4)	3 (8.8)	0.0029**
SCAs	27 (79.4)	8 (23.5)	<0.0001**
chtb/chrb	25 (73.5)	14 (41.1)	0.0136**
fra	16 (47.1)	10 (29.4)	0.2118
fra(9)(q12)	32 (94.1)	17 (50)	<0.0001**
1qh+	20 (58.8)	4 (11.8)	<0.0001**
9qh+	8 (23.5)	4 (11.8)	0.3405
inv(9)	7 (20.5)	1 (2.9)	0.5118
16qh+	9 (26.4)	0 (0)	0.0021**
Total	34	34	

*Statistically significant difference relative to unexposed group at *p* ≤ 0.05.

**Statistically significant difference relative to unexposed group at *p* ≤ 0.01 (Fisher’s exact test).

M, monosomies; T, trisomies; SCAs, structural chromosomal alterations; chtb, chromatidic break; chrb, chromosomic break; fra, fragilities; fra(9)(q12), fragility in the long arm of chromosome 9, region 1 and band 2; 1qh+, heterochromatin increased on long arm of chromosome 1; 9qh+, heterochromatin increased on long arm of chromosome 9; inv(9), inversion of chromosome 9; 16qh+, heterochromatin increased on long arm of chromosome 16; SD, standard deviation.

Within the numerical alterations, in the exposed group, monosomies (94.1%) were observed more frequently than trisomies (76.4%) (Figure 1 and Table 2). The chromosomes with the highest frequency of monosomies were the chromosomes X in 11 (32.35%) exposed, and chromosome 20 in 15 (44%) exposed. Within the trisomies, marker chromosomes were observed with a higher frequency in 21 exposed (61.76%), followed by trisomy of chromosome 22 in 9 exposed (26.47%), and trisomy of X chromosome in 7 exposed (20.58%).

Numerical chromosomal alterations were also identified in the unexposed group, where monosomies (38.2%) were observed more frequently than trisomies (8.8%) (Figure 1 and Table 2). Among the monosomies, the most frequent was the monosomy of the X chromosome observed in 8 individuals (23.52%), followed by monosomy of chromosome 2 (11.7%) in 6 (17.6%) unexposed individuals, monosomy of chromosome 12 (11.7%) in 4 (11.7%) unexposed individuals, and monosomy of chromosome 13 (11.7%) in 4 (11.7%) unexposed individuals.

**FIGURE 2:**
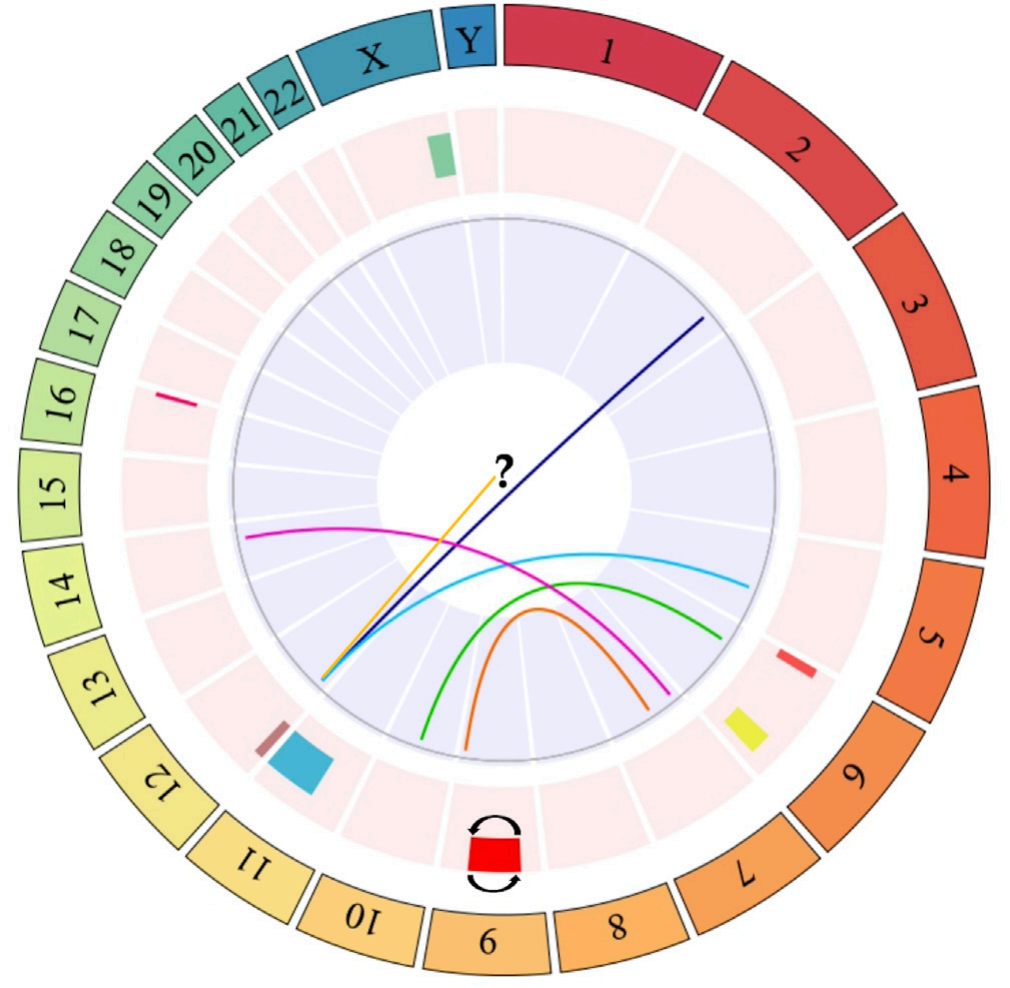
Circos plot of specific chromosomal regions commonly altered in more than one exposed individual. The outer ring indicates the number of the chromosome. The next ring indicates chromosomal abnormalities affecting only one chromosome, or where only one chromosome was identified. These alterations include: del(X)(q25) (green bar), del(6)(p23) (red bar), del(6)(q25) (yellow bar), inv(9)(p21q22) (red bar with reverse lines), del(11)(q11) (light blue bar), add(12)(p13) (yellow line), del(12)(p13) (purple bar), and del(16)(q24) (fuchsia line). The last ring (in the center of the circos plot) indicates chromosomal alterations involving more than one chromosome. These alterations include: t(2;12)(q33;p13) (dark blue line), t(5;12)(q23;p13) (light blue line), t(6;10)(p23;q22) (green line), t(7;9)(p22;q34) (orange line), t(7;14)(p22;q12) (purple line). The question mark (?) indicates additional material of unknown origin (add) attached to the short arm of chromosome 12 [add(12)(p13)]. Dark blue, light blue, green, orange, and purple links within the circos plot show translocations. The circos plot was designed in the statistical software R using the BioCircos library, later it was edited in the power point software to add some symbols that represent some alterations, which are not found in the aforementioned library.

Regarding SCAs, these were observed in the 79.41% of the exposed individuals, and in the 23.5% of unexposed individuals (Figure 1 and Table 2). A total of 88 SCAs were observed in the exposed group, being the most frequent the deletions (del) (37.5%), followed by translocations (t) (14.77%) and additional material of unknown origin (add) (9.09%). Other structural alterations observed less frequently include derived chromosomes (der) (7.95%), inversions (inv) (6.81%), dicentric chromosomes (dic) (2.27%), duplications (dup) (1.13%), isochromosomes (i) (1.13%) and ring chromosomes (r) (1.13%). The chromosomes most frequently involved in SCAs were chromosomes 4, 7, and 9, followed by chromosomes 6, X, 2, and 12. While, the chromosomes least involved in SCAs were the chromosomes Y, 14, 15, and 19. No SCAs were observed affecting chromosomes 20 and 21. The following alterations: inv(9)(p21q21), del(X)(q25), del(6)(q25), del(11)(q11) and del(16)(q24) were observed in more of one exposed (Figure 2). Regarding specific altered chromosomal regions, we observed that chromosomal regions 6p23, 7p22, and 12p13 were commonly altered in more than one (1) exposed (E3, E21, E25, E32, E34, and E35) (Figure 2).

In the unexposed group were observed a total of 13 SCAs being the most frequent the deletions (del) (50%), followed by translocations (t) (16.7%). Other less frequently observed SCAs include inversions (inv) (8.3%), derived chromosomes (der) (8.3%) and duplications (dup) (8.3%). In addition, a higher frequency of non-clonal SCAs was identified in the both groups, being these higher in the unexposed group.

With regard fragilities (fra), a higher frequency of these were found in the exposed group (625 fragilities) compared with the unexposed group (97 fragilities) (Figure 1). In both groups, many of the fragilities were non-clonal. In addition, a total of 107 chromosomal (chrb) and/or chromatic (chtb) breaks were observed in the exposed group in comparison with 26 chrb and/or chtb observed in the unexposed group (Figure 1). In the exposed group, the chromosomal and/or chromatic breaks chtb(1)(q21), chtb(1)(q10), chrb(3)(p14), chtb(3)(p21), chtb(5)(q31), chtb(6)(p21), chrb(9)(q12), chtb(12)(q15), chtb(12)(q13), chtb(13)(q31) and chtb(19)(p10) were observed in more than one (1) exposed. Comparison of the presence of CVs, chrb/chtb, NCAs and SCAs, between exposed and unexposed groups (Table 2), and between paired exposed/unexposed individuals (Table 3) showed statistically significant differences (*p* ≤ 0.001***; Fisher’s exact test, and *p* ≤ 0.05*, respectively). Although in all cases no statistically significant differences were observed between the exposed and unexposed individuals, the frequency of CVs, chrb/chtb, NCAs and SCAs was higher in the exposed group, evidencing chromosomal damage due to exposure to pesticides.

**TABLE 3:** Frequency (n) and percentage (%) of chromosome variants (CVs) and chromosomal alterations (CAs) identified in paired exposed/unexposed individuals.

No	Exposed		Unexposed		*p*
	n	%	n	%	
1	138	9.24	14	1.31	0.01**
2	89	5.96	6	0.56	0.02*
3	109	7.30	14	1.31	0.06
4	109	7.30	7	0.65	0.01**
5	65	4.35	21	1.97	0.68
6	60	4.01	4	0.37	0.12
7	105	7.03	10	0.94	0.06
8	43	2.88	14	1.31	0.62
9	4	0.26	2	0.18	0.99
10	5	0.33	6	0.56	0.99
11	3	0.20	5	0.47	0.99
12	54	3.61	28	2.63	0.99
13	62	4.15	10	0.94	0.36
14	5	0.33	8	0.75	0.99
15	15	1.00	8	0.75	0.99
16	0	0	5	0.47	0.99
17	42	2.81	8	0.75	0.24
18	101	6.76	5	0.47	0.01**
19	52	3.48	4	0.37	0.12
10	45	3.01	0	0	0.24
21	31	2.07	1	0.09	0.49
22	43	2.88	10	0.94	0.62
23	14	0.93	0	0	0.99
24	28	1.87	7	0.65	0.49
25	30	2.00	2	0.18	0.49
26	23	1.54	1	0.09	0.49
27	72	4.82	0	0	0.05*
28	13	0.87	2	0.18	0.99
29	26	1.74	1	0.09	0.49
30	16	1.07	2	0.18	0.99
31	6	0.40	0	0	0.99
32	12	0.80	2	0.18	0.99
33	16	1.07	1	0.09	0.99
34	35	2.34	1	0.09	0.49

*Statistically significant difference relative to the unexposed group at *p* ≤ 0.05.

**Statistically significant difference relative to the unexposed group at *p* ≤ 0.01 (Fisher’s exact test).

The total number of metaphases analyzed in the exposed group was 1493, while in the unexposed group (control) it was 1061.

The evaluation of the effect of smoking and alcohol consumption as confounding factors on the frequency of CV, chrb, chtb and CCA and NCCA (numerical and structural chromosomal alterations) in all study subjects, allowed us to conclude that none of these (alcohol consumption, smoking) increases the frequency of CVs and CAs in any of the groups studied, exposed and unexposed (Table 1 and Supplementary Table S1).

### FISH Results

**FIGURE 3:**
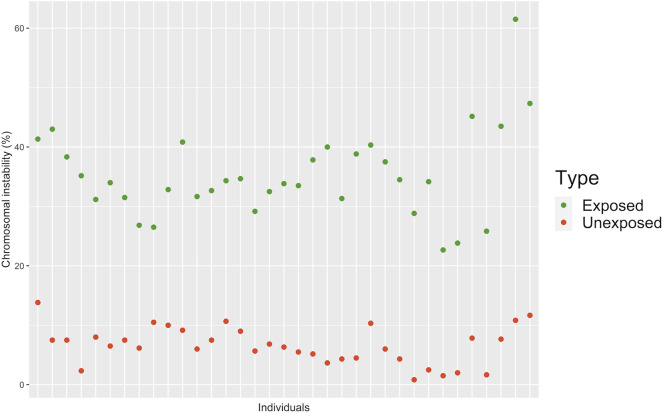
Percentage of CIN assessed by FISH in 100 interphasic nuclei in the exposed and unexposed groups. According to the level of CIN, each exposed and unexposed individual was classified as having low CIN (CIN < 25%) or high CIN (CIN ≥ 25%).

**FIGURE 4:**
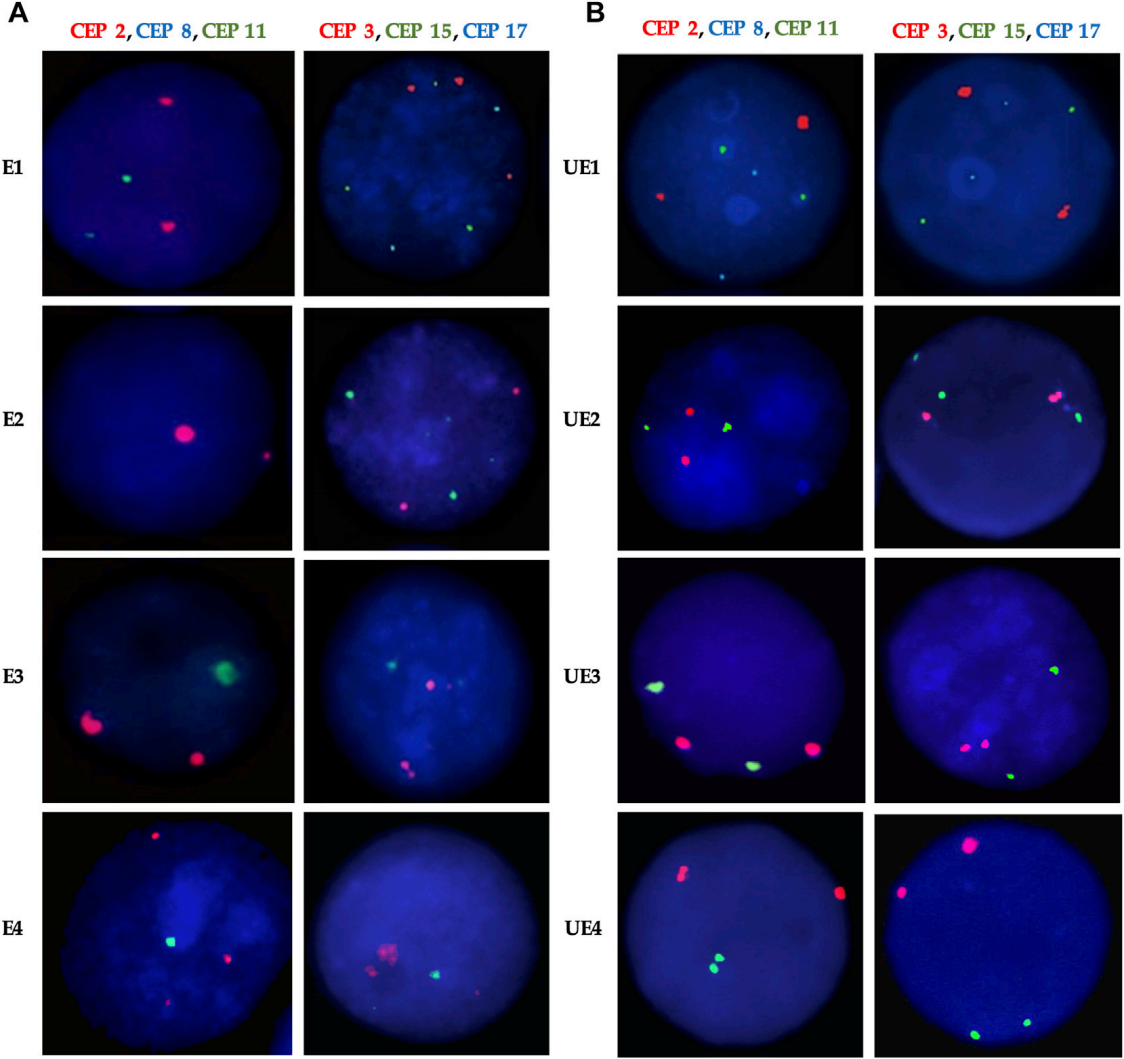
Representative FISH images for **(A)** Exposed and **(B)** Unexposed individuals. Three-color FISH was performed on nuclei spreads for chromosomes 2, 8, and 11 and, chromosomes 3, 15, and 17 using centromeric probes (CEP) labeled with different spectrum colors: spectrum orange for CEP2 and CEP3; spectrum aqua for CEP8 and CEP17; and spectrum green for CEP11 and CEP15**.** Interphase nuclei at each treatment time point are indicated. E, Exposed; UE, Unexposed individuals.

We assessed CIN in 100 interphase nuclei and some metaphases by using centromeric FISH. Exposed individuals showed a high CIN (≥22.67%) compared with a low CIN (≤13.83%) observed in unexposed individuals (Figures 3, 4, and Supplementary Table S2). More specifically, in exposed individuals, CIN ranged between 22.67 and 47.33%, while in non-exposed individuals, CIN ranged between 0.83 and 13.83% (Figures 3, 4).

The mean CIN was 34.57% ± 6.03 for exposed, and 6.48% ± 3.13 for unexposed. Student’s t-test showed statistically significant differences (*p* < 0.001**) between the CIN of the exposed and unexposed individuals. These results suggest that pesticides can induce aneuploidy, which is indicative of numerical CIN.

**FIGURE 5:**
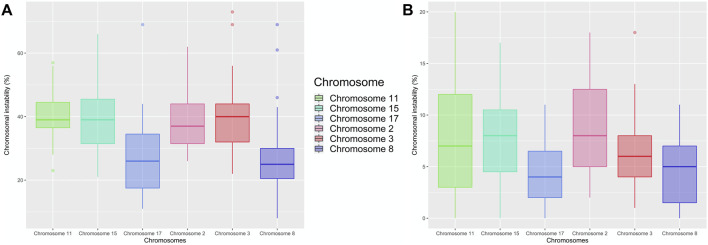
Percentage of CIN in the Exposed **(A)** and Unexposed **(B)** groups. According to the level of CIN, each chromosome was classified as having low CIN (CIN < 25%) or high CIN (CIN ≥ 25%). The most stable chromosomes for exposed individuals were chromosome 8 and 17, and the most unstable chromosomes were chromosome 2 and chromosome 15. While for unexposed individuals, the most stable chromosome were chromosomes 8 and 17 as well, and the most unstable chromosome was chromosome 3.

In order to determine the most stable chromosomes in the groups studied (exposed and unexposed), we carried out the Kruskal–Wallis test. This test showed in the exposed group, a statistically significant difference (*p* < 0.001***) between chromosomes 2, 3, 11, and 15, and chromosomes 8 and 17, with chromosomes 8 and 17 being the most stable. For the unexposed group, statistically significant differences were also observed (*p* < 0.001***) between the chromosomes 3, 11, and 15; the chromosomes 2, 11, and 15 and the chromosomes 8 and 17, with chromosomes 8 and 17 being the most stable, similar to what was observed in the exposed group (Figure 5).

### Clonal Heterogeneity

**FIGURE 6:**
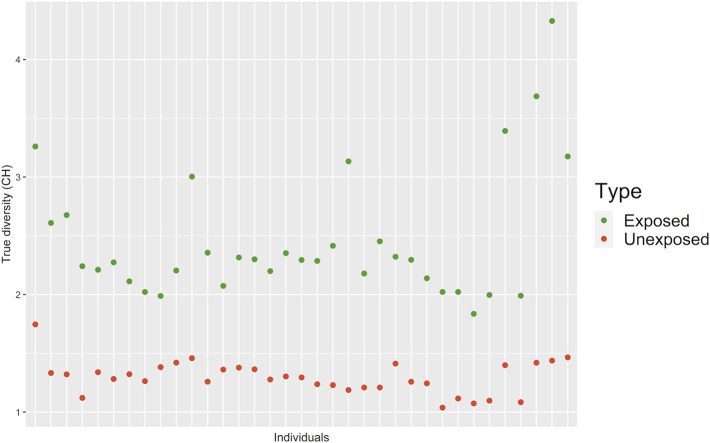
Clonal heterogeneity (CH) determined by True Diversity (TD) for exposed and unexposed groups. Values below 1.5 were considered indicative of low CH, values between 1.6 and 2 were considered indicative of intermediate CH; and values higher than 2 were considered indicative of high CH.

In order to determine the CH in the both groups, two different but related indices were used, the SDI and true diversity index (TD), which integrate the number and abundance of cell clones in each individual (exposed and unexposed) according to published methods (Maley et al., 2006; Jost and Gonzá lez-Oreja, 2012). CH was 1.99 higher in the exposed group than in the unexposed group. Significant statistical differences between exposed and unexposed groups for both, TD (*p* < 0.001***; Non-parametric Mann Whitney Wilcoxon) and the SDI (*p* < 0.001***; Non-parametric Mann Whitney Wilcoxon) were observed (Figure 6, Supplementary Table S2, Supplementary Figure S1).

Likewise, CH was also determined for each of the chromosomes studied in each group. For both groups, statistically significant differences were observed, for both TD (*p* < 0.001***; Kruskal–Wallis test) (Supplementary Figure S2) and for SDI (*p* < 0.0016***; Kruskal–Wallis test) (Supplementary Figure S3), and between the group of chromosomes 2, 3, 11, and 15 and the group of chromosomes 8 and 17, being chromosomes 8 and 17, those with the lowest CH.

### Correlation of Variables

**FIGURE 7:**
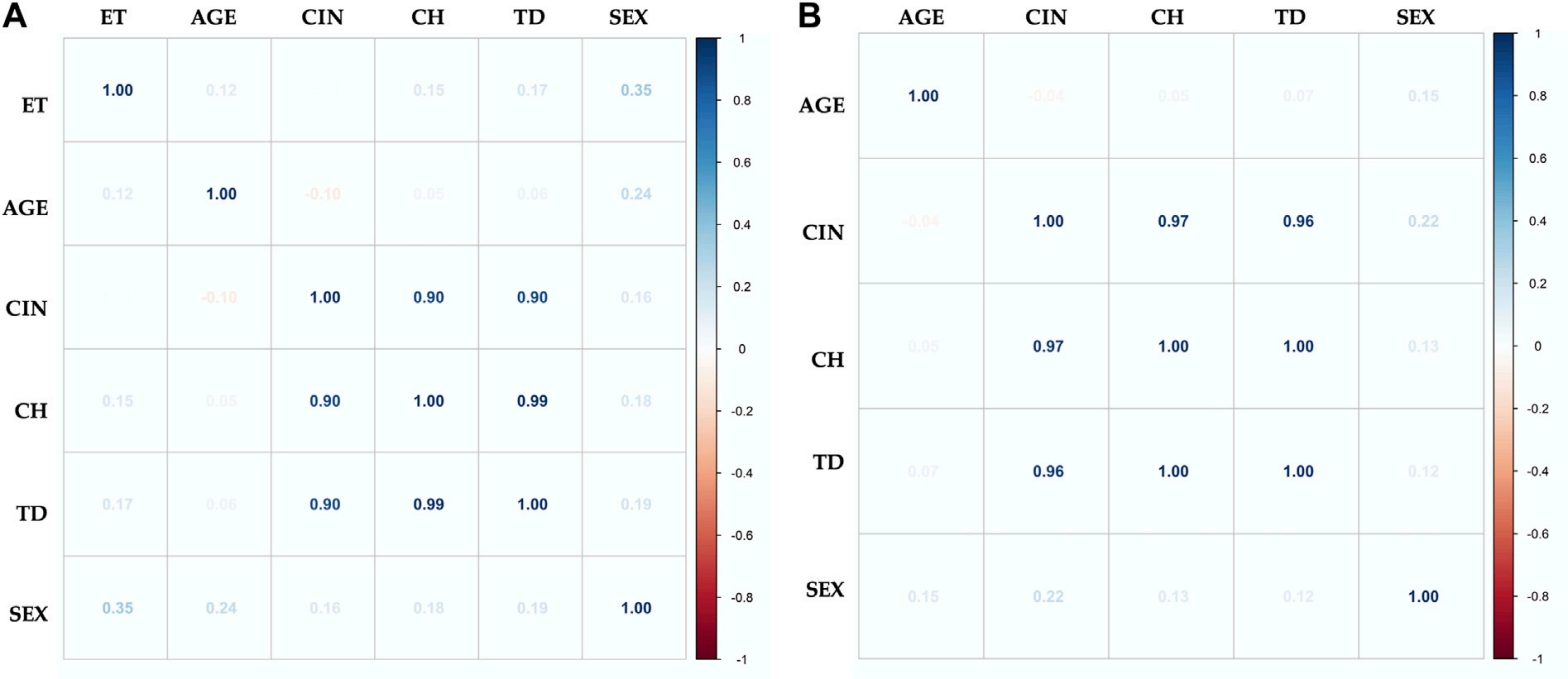
Multivariate analysis with Pearson correlation coefficient for **(A)** Exposed and **(B)** Unexposed groups. Values greater than 0.5 are indicative of a statistically significant correlation. No linear correlation was found between chromosomal instability (CIN), clonal heterogeneity (CH) and true diversity index (TD) with any of the variables studied: time of exposure to pesticides (TE), age, and sex.

In order to establish in both groups, exposed and unexposed, the existence of associations between the levels of CIN and CH (TD), with variables such as age, sex, and time of exposure (TE) to pesticides (only in the exposed group), we perform multivariate analysis using the Pearson correlation coefficient. In both groups, a strongly positive relationship was found between the CIN and CH. However, no linear correlation was found between CIN and CH with any of the variables studied (age, sex, and TE to pesticides) (Figure 7). The variables smoking and drinking habits, were not evaluated due to the low prevalence reported by the two groups.

## Discussion

Pesticides are a heterogeneous category of chemicals specifically designed for pest control. Although its application continues to be the most effective method for protecting plants against pests, its use has been associated with harmful effects on the health of the people involved in its regular and extensive use. In fact, it has been indicated that farmers occupationally exposed to pesticides during spraying activities are more prone to genotoxicity than those not exposed. In this regard, some studies have identified chromosomal damage related to pesticide exposure in various populations, however, in these studies, information on the type and frequency of specific CAs and CVs, as well as the level of CIN and CH induced by the exposure to pesticides is scarce. In fact, one of the few available studies indicating the type and frequency of specific chromosomal alterations induced by pesticide exposure was reported by us, in a small group of exposed (five exposed) (Cepeda et al., 2020). In this study, we observed a significant increase in clonal and non-clonal chromosomal alterations in individuals exposed to pesticides compared to unexposed individuals (Cepeda et al., 2020). Considering the importance of our previous findings in the identification of cytogenetic biomarkers for the monitoring of exposed populations, we decided to conduct a new study with a greater number of individuals exposed to pesticides.

Our results indicate that occupational exposure to pesticides was associated to a significant increase in CIN, in agreement with previous reports indicating DNA damage in populations occupationally exposed to pesticides (Grover et al., 2003; Castillo-Cadena et al., 2006; Wilhelm et al., 2015). Our results show that individuals exposed to pesticides have a high frequency of CAs, CVs, CIN, and CH compared to low frequency observed in unexposed individuals. The mean number of CVs and CAs observed in the exposed individuals was five times higher than in the unexposed individuals. Numerical and structural chromosomal alterations were higher and with a statistically significant prevalence in the exposed group. These findings suggest a possible cytogenetic effect of pesticides on occupationally exposed individuals.

Regarding the numerical alterations identified in both study groups, a high frequency of aneuploidy, was observed in the exposed group compared to the unexposed group. Aneuploidy refers to the gain and/or loss of complete chromosome, which can be stable or unstable. Unstable aneuploidy (cell-to-cell variation in chromosome number) may favor the simultaneous growth of various cellular subpopulations leading to genomic heterogeneity (Bolt et al., 2004; Gagos and Irminger-Finger, 2005; Geigl et al., 2008; Tanaka and Hirota, 2016; Vargas-Rondon et al., 2017). Even though the mechanisms by which pesticides induce aneuploidy are not fully understood, it has been suggested that they can lead to chromosomal nondisjunction, and thus to the loss or gain of entire chromosomes, by interacting with a variety of cellular processes including, the alteration in the formation of chromosomal microtubules responsible for segregation of genetic material during cell division (Lushchak et al., 2018); the synthesis, division and functioning of centrioles, polar bodies and spindle fibers (Zijno et al., 1996); the assembly and functioning of the kinetochore proteins (Parry et al., 2002), and the centrosome activity and the modification of centromeres (Renzi et al., 1996; Mattiuzzo et al., 2006).

In addition to the numerical alterations, we also observed in the exposed group, high frequency of structural chromosomal alterations. The chromosomes most frequently involved in structural alterations were chromosomes 4, 7, and 9, followed by chromosomes 6, X, 2, and 12. Regarding specific chromosomal regions, we observed that chromosome regions 6p23, 7p22, and 12p13 were involved in more than one chromosomal alteration and in more than one (1) exposed. It should be noted that these affected chromosomal regions have been implicated in the development of various types of cancer (Table 4), evidencing the importance of their evaluation and/or identification in people exposed to genotoxics.

**TABLE 4:** Chromosomal regions involved in chromosomal alterations in the exposed group and associated with the development of various types of cancer.

Type	Associated disease	Tumor site	Band	Abnormality	References
Unbalanced	Acute lymphoblastic leukemia/lymphoblastic lymphoma		Xq25	del(X)(q25)	Heerema et al. (1992), Wuicik et al. (2007), Nayebbagher et al. (2020)
Unbalanced	Adenocarcinoma	Stomach, Breast	Xq25	del(X)(q25)	
Unbalanced	Acute lymphoblastic leukemia/lymphoblastic lymphoma, Acute myeloid leukemia		6p23	del(6)(p23)	Sawyer et al. (1996), Takeshita et al. (2004), Anwar Iqbal et al. (2006)
Unbalanced	Astrocytoma, grade III-IV/Glioblastoma	Brain	6p23	del(6)(p23)	
Unbalanced	Multiple myeloma		6p23	del(6)(p23)	
Unbalanced	Acute lymphoblastic leukemia		6q25	del(6)(q25)	Rogatto et al. (1993), Debiec-Rychter et al. (1995), Tibiletti et al. (1996), Gladstone et al. (1998), Tibiletti et al. (2000), Tibiletti et al. (2003), Amare Kadam et al. (2004), Cerretini et al. (2006), Travella et al. (2013), Sawyer et al. (2014)
Unbalanced	Adenocarcinoma	Breast, Ovary	6q25	del(6)(q25)	
Unbalanced	Astrocytoma, Glioblastoma	Brain	6q25	del(6)(q25)	
Unbalanced	Benign epithelial tumor	Breast	6q25	del(6)(q25)	
Unbalanced	Burkitt lymphoma		6q25	del(6)(q25)	
Unbalanced	Ependymoma	Cerebellum	6q25	del(6)(q25)	
Unbalanced	Multiple myeloma		6q25	del(6)(q25)	
Unbalanced	Retinoblastoma	Eye	6q25	del(6)(q25)	
Unbalanced	Teratoma	Testis	6q25	del(6)(q25)	
Balanced	Acute lymphoblastic leukemia		7p22	t(7;14)(p22;q11)	Prigogina et al. (1988), Olsson et al. (2018)
Balanced	Chronic myeloid leukemia		7p22	t(7;9;22)(p22;q34;q11)	El-Zimaity et al. (2004), Lee et al. (2012), Issa et al. (2017)
Unbalanced	Acute myeloid leukemia		9p21	46,XX,inv(9)(p21q22)	Nahi et al. (2008)
Unbalanced	Acute myeloid leukemia, Chronic lymphocytic leukemia		11q11	del(11)(q11)	Rigolin et al. (1997), Mandahl et al. (2000), Wang et al. (2001), Pantou et al. (2005), Gabrea et al. (2008), Rayeroux and Campbell, (2009), Campioni et al. (2012)
Unbalanced	Leiomyosarcoma	Soft tissue	11q11	del(11)(q11)	
Unbalanced	Multiple myeloma		11q11	del(11)(q11)	
Unbalanced	Acute lymphoblastic leukemia, Acute myeloid leukemia		12p13	add(12)(p13)	Pejovic et al. (1991), Presti et al. (1991), Bardi et al. (1993), Rodriguez et al. (1993), Hoogerwerf et al. (1994), Testa et al. (1994), Pandis et al. (1995), Bridge et al. (1997), Feder et al. (1998), Smolarek et al. (1999), Teixeira et al. (2001), Kirkhorn and Schenker, (2002), Lloveras et al. (2004), Karst et al. (2006), Kowalski et al. (2007), Al-Bahar et al. (2010), Hong et al. (2016), Ashok et al. (2017), Ampatzidou et al. (2018)
Unbalanced	Adenocarcinoma	Lung, Pancreas, Large intestine, Kidney, Breast Ovary	12p13	add(12)(p13)	
Unbalanced	Osteosarcoma	Skeleton	12p13	add(12)(p13)	
Unbalanced	Teratoma (mature and immature)	Testis	12p13	add(12)(p13)	
Unbalanced	Liposarcoma, dedifferentiated	Intraabdominal	16q24	del(16)(q24)	Pedersen et al. (1986), Macarenco et al. (2006)
Unbalanced	Malignant melanoma	Skin	16q24	del(16)(q24)	

The implications of numerical and structural chromosomal alterations in the development of diseases could be due to the fact that chromosomal alterations can lead to altered expression of genes (proto-oncogenes and tumor suppressor genes) and variable protein concentrations, which control cell cycles and differentiation processes, and in turn may cause an unbalance at the cellular level with serious biologic consequences (Paz-y-Miño et al., 2002).

In addition to numerical and structural chromosomal alterations, a high frequency of fragilities (fra), chrb and chtb, was observed in the exposed group compared to the low frequency of the same observed in the unexposed group. Fragilities may be resulted from single-strand DNA breaks (Glover, 1998), which if not repaired, may lead to chromosome damage such as intrachromosomal gene amplification (Coquelle et al., 1997), sister chromatid exchanges (Glover and Stein, 1987), deletions (Durkin and Glover, 2007), duplications (Hellman et al., 2002) and translocations (Re et al., 2006), among other, all of them associated with the development of cancer (Debacker and Kooy, 2007; Vincent-Salomon et al., 2013). Regarding chrb and chtb, both are chromosomal aberration that involves single and/or double stranded DNA breaks. Double-stranded DNA breaks can be induced by reactive oxygen species (ROS), which are highly reactive molecules involved in various cellular processes, causing fragmentation and oxidation of nucleic acids, proteins and lipids (Kaur and Kaur, 2018), and also associated with the exposure to pesticides (Hilgert Jacobsen-Pereira et al., 2018; Kaur and Kaur, 2018; Shah et al., 2020). Further, increased oxidative stress and ROS production due to pesticide use, has been associated with reproductive disorders in women, including cycle defects, folliculogenesis, follicular atresia, implantation defects, miscarriages and endometriosis (Bhardwaj et al., 2020). The presence of chrb and chtb in the exposed group, could predispose a greater risk to develop complex chromosomal rearrangements such as translocations, inversions, dicentric chromosomes, deletions and duplications, thus evidencing the high CIN associated with exposure to pesticides observed in our study.

Although chromosomal heteromorphisms (observed by us in higher frequency in the exposed group) are considered normal chromosomal variants, variations in size and location of the major heterochromatic regions (1qh, 9qh, 16qh) have particularly been implicated in various cancers and leukemias (Wyandt HE, 2004). For instance, Atkin (1977) first suggested susceptibility to malignancy associated with heteromorphisms in chromosome 1. In addition, rearrangements in the vicinity of the centromere of chromosome 1 have been reported as over-represented in many types of human cancers (Ji et al., 1997). Subsequent observations were reported for chromosomes 1, 9, and 16 and the Y chromosome and include observations of increased or decreased length, striking size differences between homologs (asymmetry), and pericentric inversions in heterochromatic regions. For example, an increase in heterochromatin of chromosome 16 was observed in couples with a stillborn or a malformed child (Buretic-Tomljanovic et al., 1997).

In our study, the observation of a higher frequency of chromosomal variants in the exposed group is noteworthy and could have important implications in the monitoring of populations exposed to pesticides. The above, considering not only the findings previously described, but additional studies that indicate that not all chromosomal variants involve only heterochromatin. Indeed, rearrangements in the pericentromeric region of chromosome 1 or 16, common in various types of cancers, are known to involve particular oncogenes that are close to the pericentromeric regions (Mugneret et al., 1995; Tse et al., 1995). The inversions or insertions of these genes in heterochromatin regions could possibly play a role in the activation or deactivation of these genes through positional effects (Wyandt HE, 2004).

To highlight that, while the numerical chromosomal alterations observed in the exposed group were mainly clonal (CCAs), the structural chromosomal alterations were non-clonal (NCCAs). CCAs and NCCAs can lead to clonal selection and to the expansion of chromosomal alterations, thus increasing overall heterogeneity. Both clonal selection and heterogeneity reflect the instability of the system and could lead to development of diseases by increasing the diversity of the cell population. Even though, NCCA have been considered as *in vitro* culture artifact because they are non-recurrent abnormalities, they have acquired great importance in recent years, given their correlation with both CIN and genomic diversity (heterogeneity) and with their involvement in the development of diseases (Rangel et al., 2017; Vargas-Rondon et al., 2017), so identifying and reporting these alterations is clinically relevant. In fact, NCCAs are the key elements that initiate the formation of CCAs (discontinuous interrupted phase) and provide the basis for the formation of diverse populations with clonal changes (gradual phase), thus leading to CIN and CH (Rangel et al., 2017; Vargas-Rondon et al., 2017). In fact, some authors have suggested that although NCCAs are not stable and cannot survive, they provide the genetic variation necessary for macrocellular evolutionary selection and for CH (Liu et al., 2014). A heterogeneity-generating event that could lead to nonclonal structural chromosomal alterations and clonal aneuploidy is the break-fusion-bridge (BFB) cycle. BFB cycles may lead to a considerable intercellular heterogeneity participating in the formation of dicentric chromosomes, ring chromosomes and/or acentric chromosomes, among others (Gisselsson et al., 2000). At anaphase, such rearranged chromosomes frequently fail to segregate in an orderly manner, instead forming nucleoplasmic bridges (NPB) between the spindle poles (Gisselsson et al., 2001). As result of the formation of NPB, the lagging chromosome may be lost, form a micronucleus (MN), or be randomly incorporated into either of the daughter nuclei, conducing to clonal aneuploidy. Moreover, at the anaphase-telophase transition, these NPB may subsequently break, resulting in novel SCAs in the daughter cells (Gisselsson et al., 2001; Fenech et al., 2011), thus favoring the presence of non-clonal alterations. To highlight that these abnormal nuclear shapes (NPB and MN) have been considered as common features of a wide variety of unstable cells (Gisselsson et al., 2001; Caradonna, 2015). Overall, our results suggest that SCAs appear to play a major role in conferring genetic heterogeneity (NCCAs), potentially surpassing the variability observed at the numerical level (CCAs).

Additionally, the high frequency of CIN and CH observed in this study by using GTG banding was confirmed by using FISH. FISH allows detecting the appearance of CIN, CH and clonal evolution before it is detected in metaphases. For instance, have been indicated that although the presence of a Philadelphia (Ph) chromosome was identified through the use of banding cytogenetics in peripheral blood and bone marrow samples from patients with chronic myeloid leukemia, the use of FISH assays allowed to identify a certain percentage of cells with an additional Ph + chromosome, not identified by banding cytogenetics (Bentz et al., 1994; Buno et al., 1998), which confirms the usefulness of FISH assays to identify CIN, CH and clonal evolution in peripheral blood samples.

The results obtained in our study using FISH, suggest a negative effect of occupational pesticide exposure on the stability of the chromosomes. FISH results showed that individuals exposed to pesticides have a high level of CIN (≥22.67%) compared to low CIN (≤13.83%) observed in unexposed individuals. The CIN level was 33.5 times higher in the exposed group than in the unexposed group. In addition, we also observed differences in CH levels, being it statistically higher in the exposed group than in the unexposed group. These results suggest that the high CH observed in the exposed individuals, could be the result of the high levels of CIN also presented in these individuals.

To highlight that, CH has not been evaluated in previous studies of occupational exposure to genotoxic agents, therefore, the results of our study are very important, since they show that exposure to pesticides induces CIN and CH, which in addition to reflecting the instability of the system, could predispose cells to acquire additional CIN and, therefore, to a higher risk of malignant transformation (Zhang et al., 2011; Cepeda et al., 2020). In fact, CIN has been recognized as a source of genetic variation that leads to CH, thus favoring the adaptation of cells to stressful environments and the possibility of the development of diseases, mainly cancer (Dayal et al., 2015).

In order to quantify CH, diversity measures adopted from ecology and evolution have been applied, including the SDI, which has been widely used to determine CH in cell lines (Lengauer et al., 1997; Munro et al., 2012). However, some ecologists have suggested that although the SDI is effective for measuring diversity, it does not represent diversity per se, and its misuse could lead to confusion (Jost and Gonzá lez-Oreja, 2012). Thus, we suggest the use of TD as an indicator of CH since it allows us to obtain a more realistic value of heterogeneity.

In line with previous studies (Pastor et al., 2003; Sailaja et al., 2006; Benedetti et al., 2018) we did not find associations between CIN and CH levels with variables such as sex, age, and exposure time (ET). This could suggest that the chromosomal damage induced by pesticides is independent of sex, age, and ET, and highlights the importance of identifying biomarkers that allow monitoring of exposed populations. One such biomarker is the evaluation of CIN and CH by FISH, using centromeric probes for chromosomes 2, 3, 8, 11, 15, and 17. In fact, according to our results, chromosomes 8 and 17 could be excellent biomarkers of chromosomal stability, since these chromosomes did not show great variations in the groups studied. The stability observed in chromosomes 8 and 17 could make it possible to detect damage to the genetic material by observing variations in the number of copies of these chromosomes.

Since most of the farmers who participated in our study were exposed to complex and variable mixtures of pesticides, it is not possible for us to establish whether the CAs, CVs, CIN, and CH observed in the exposed individuals are due to a single pesticide. In fact, even where associations have been seen or suspected, identifying the specific agent responsible has been difficult for a variety of reasons, including the variable exposure levels, and concurrent exposure to multiple pesticides. The above constitute a great problem and concern in public health, considering that some studies have indicated that mixtures of toxics can influence and even amplify the toxicity of the individual components through synergies, potentiation, antagonism, inhibition or effects additives (Mumtaz, 1995; Reffstrup et al., 2010). It is important to highlight that, although a limitation of our study was the impossibility of establishing associations between individual pesticides with the induction of chromosomal alterations (for the reasons indicated above), our results suggest the deleterious effect of the pesticide mixture on chromosomes. In this regard, few *in vitro* and *in vivo* studies have reported associations between some individual pesticides with the induction of chromosomal alterations. For instance, and with regard to the pesticides used most frequently by the exposed individuals included in our study, associations between mancozeb exposure with a significant increase in the frequencies of structural chromosomal alterations and genotoxic damage were reported (Jablonicka et al., 1989; Srivastava et al., 2012). In addition, *in vitro* studies in human lymphocytes demonstrated associations between exposure to paraquat and the production of isochromatic breaks (Jovtchev et al., 2010), as well as between high concentrations of chlorpyrifos with an increase in the number of numerical chromosomal alterations (Serpa et al., 2019), and between sublethal concentrations of profenofos with the induction of chromatid breaks and gaps (Prabhavathy Das et al., 2006). In the same way, *in vivo* cytogenetic analysis demonstrated the induction of chromosomal alterations and micronucleus (MN) formation in mouse bone marrow cells exposed to furadan (Chauhan et al., 2000). Unfortunately, despite the deleterious effect of pesticides on human health, only few studies have investigated the effect of individual pesticides on human chromosomes.

Some chemical classes of pesticides used by the exposed individuals, such as organophosphates and carbamates, have been reported to be genotoxic, generating free radicals that react with cell membranes and initiate the process of lipid peroxidation (Banerjee et al., 1999). In fact, it has been reported that mancozeb, one of the pesticides used by farmers in this study, is a carbamate fungicide commonly used for a wide spectrum of crops (especially soy) and contains a substance with important effects on human health: ethylene(bis) dithiocarbonate (EBCD). EBCD is easily metabolized into ethylene thiourea (ETU), which decreases the activity of tumor suppression proteins, thus facilitating tumor growth (George and Shukla, 2011; Paro et al., 2012). Paraquat, another of the pesticides used by farmers, besides being the second most widely used prototypical agricultural herbicide (Sabarwal et al., 2018), also been associated with an increased risk of Parkinson’s disease, with effects mainly in the liver and kidney (O'Leary et al., 2008), and with pulmonary fibrosis through the generation of ROS (Kirkhorn and Garry, 2000). Overall, pesticides have been associated with deleterious effects on the health of exposed people, including the interfere of the endocrine system and neurobehavioral development (LeBlanc et al., 1997), the development of respiratory symptoms and immunodeficiency (Hoppin et al., 2002), the development of diseases such as breast, lung and pancreatic cancer, lymphomas, among others, which generates a public health problem (Kawauchi et al., 2010; Arafa et al., 2013; Farkas et al., 2016).

The results of this study suggest that occupational exposure to pesticides is associated with CAs, CVs, CIN, and CH in somatic cells of Colombian farmers. Chromosomal damage is an important step in carcinogenesis and the development of many other diseases. Considering that CIN can predispose cells to additional chromosomal alterations (CH) and, therefore, to an increased risk of developing diseases, the monitoring of these markers (CAs, CVs, CIN, and CH) could be useful to estimate the genetic risk in populations exposed to pesticides. Our results highlight the need to develop educational programs aimed at controlling the use of these substances and implementing prevention and protection measures in exposed populations. Therefore, effective efforts are required to support and monitor populations exposed to pesticides, as well as implement more stringent guidelines that help reduce potential genotoxic harm. Further, early detection of chromosomic damage is crucial to implement the necessary measures to reduce or suppress the exposure to deleterious agent when the damage is still reversible, thus reduce the risk to suffer diseases.

## Data Availability

The original contributions presented in the study are included in the article/Supplementary Material, further inquiries can be directed to the corresponding authors.
