# Supplementary figures and images for: Profile of Chromosomal Alterations, Chromosomal Instability and Clonal Heterogeneity in Colombian Farmers Exposed to Pesticides
Source: Front Genet. 2022 Feb 24;13:820209. doi: 10.3389/fgene.2022.820209 (PMC8908452; doi:10.3389/fgene.2022.820209)

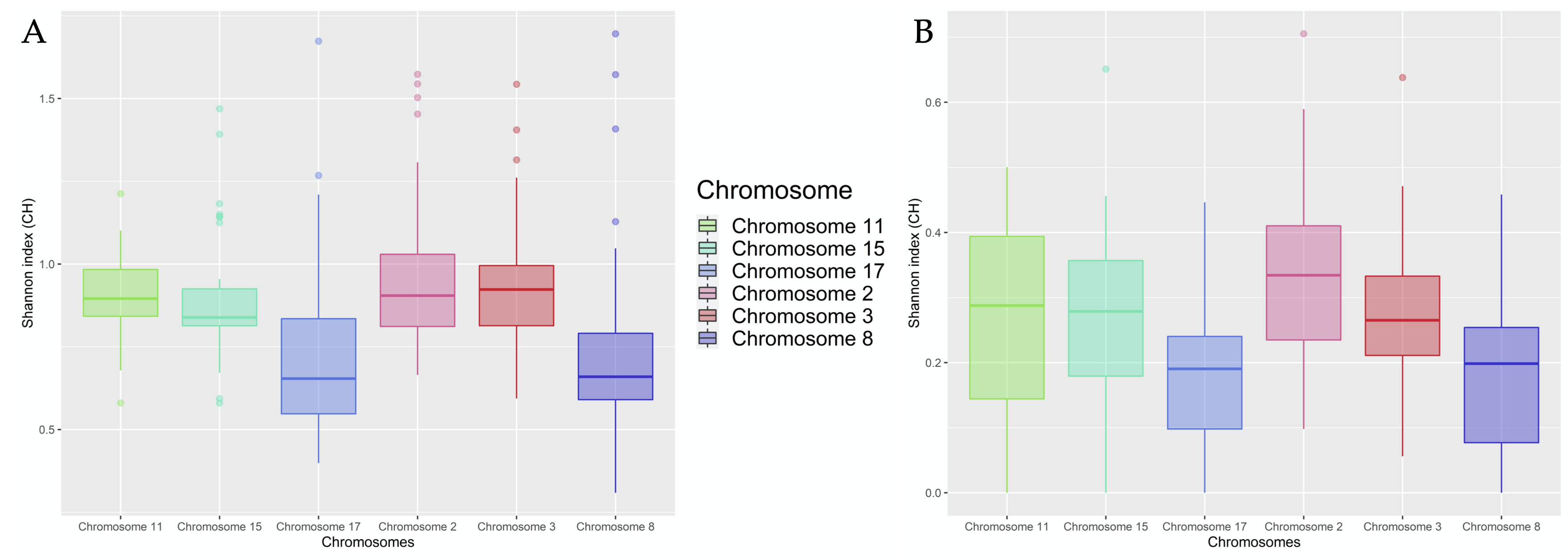

Supplement: Supplementary file 1 [file Image3.TIFF]

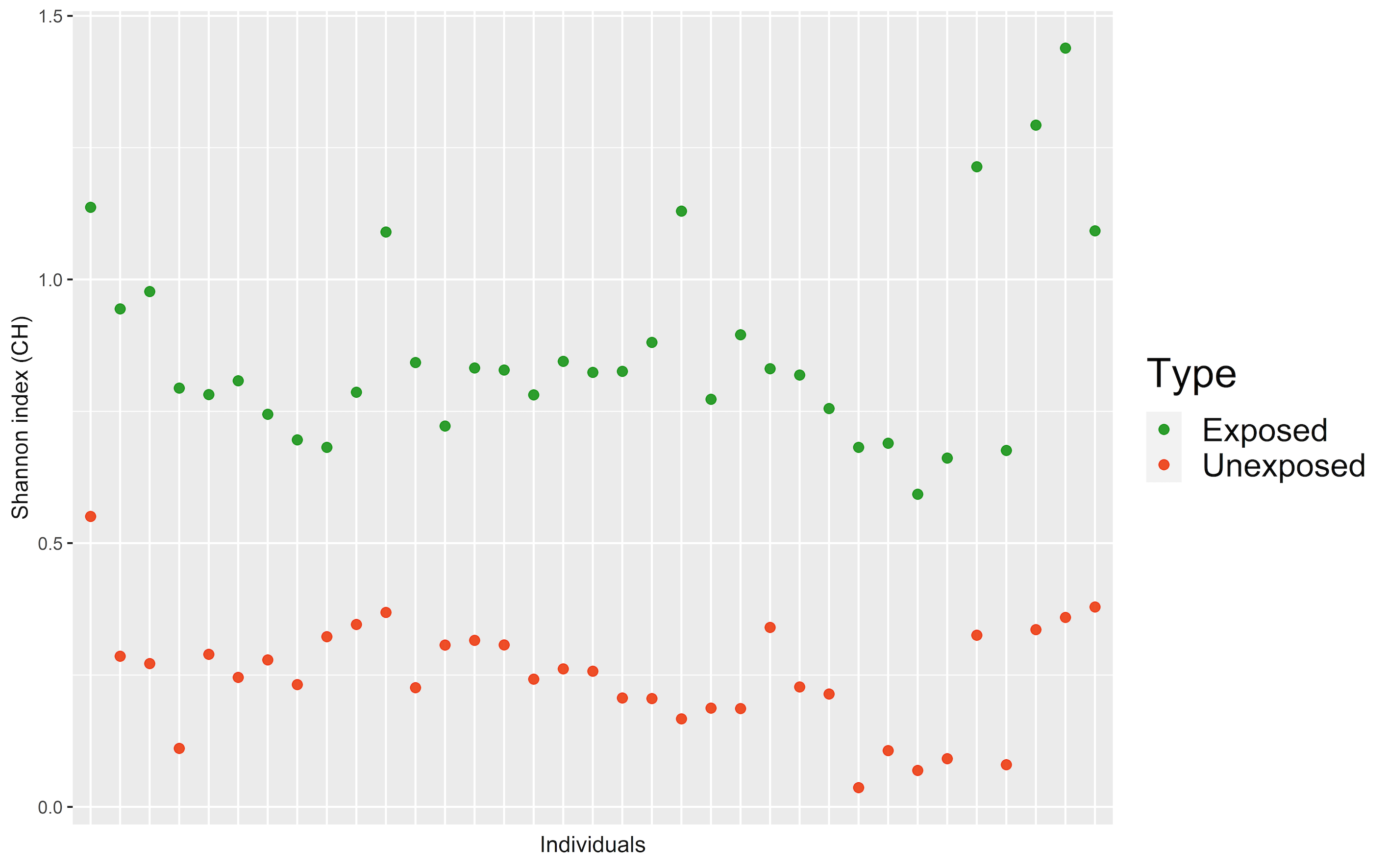

Supplement: Supplementary file 2 [file Image1.TIFF]

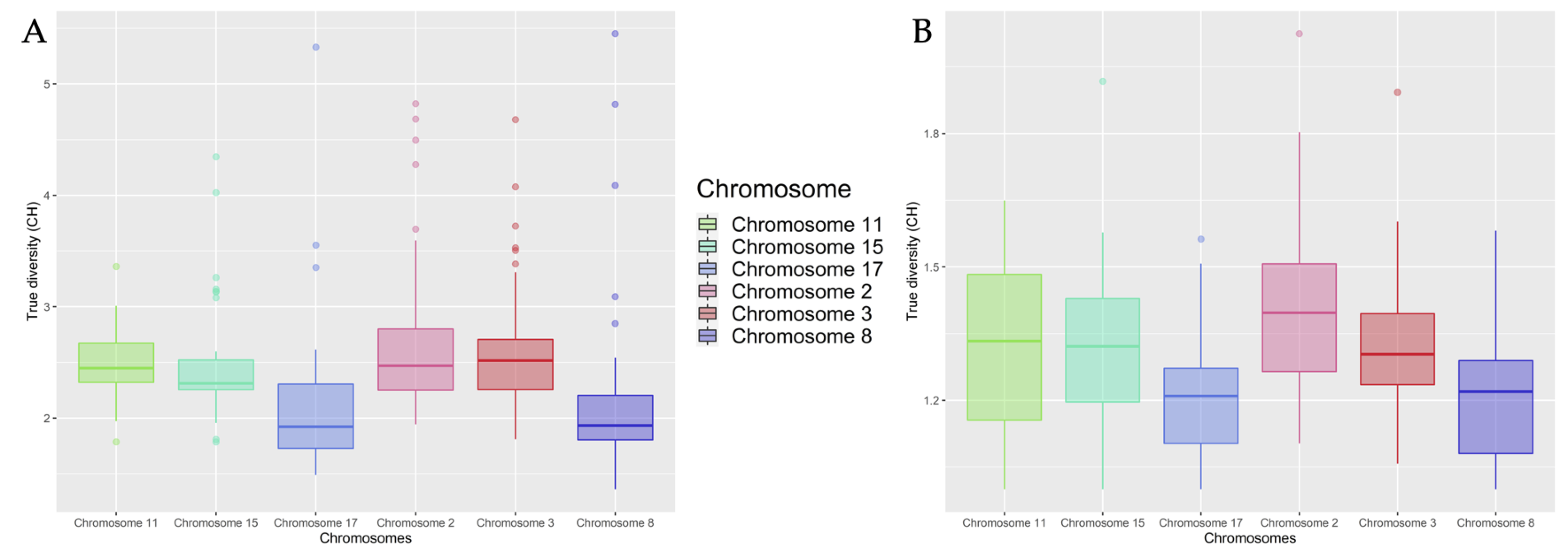

Supplement: Supplementary file 3 [file Image2.TIFF]
