# Supplementary material for: Profile of Chromosomal Alterations, Chromosomal Instability and Clonal Heterogeneity in Colombian Farmers Exposed to Pesticides
Source: Front Genet. 2022 Feb 24;13:820209. doi: 10.3389/fgene.2022.820209 (PMC8908452; doi:10.3389/fgene.2022.820209)
Supplement: Supplementary file 4 [file DataSheet1.docx]

Supplementary Material

**Supplementary Table 1**. Detailed characteristics of the groups studied

| **E** | **Age (y)** | **Exposure time (m)** | **Pesticide exposure frequency** | **Pesticide mixture and Dose (Kg/ha)** | **Number of ha sprayed per day** | **Habits** | | **UE** | **Age (y)** | **Habits** | |
| --- | --- | --- | --- | --- | --- | --- | --- | --- | --- | --- | --- |
|  |  |  |  |  |  | **S** | **D** |  |  | **S** | **D** |
| **1** | 66 | 120 | Daily | Paraquat (0.3); Mancozeb (2) | 10 - 20 | - | - | **1** | 64 | - | 1 / m |
| **2** | 63 | 60 | Once a week | Paraquat (0.3); Neguvon (0.003); Fungicide (2) | 1 | - | 1 / w | **2** | 62 | - | 1 / m |
| **3** | 52 | 48 | Once a week | Mancozeb (2); Furadan (0.72); Dimecron (0.2) | 1 | - | - | **3** | 52 | - | - |
| **4** | 51 | 126 | Once a week | Cerillo (0.552); Eltra (1.2); Forum (0.8) | 6 | - | 1 / m | **4** | 52 | - | 2 / y |
| **5** | 53 | 420 | Once a week | Mancozeb (2); Furadan (0.72); Curacron (0.4); Lorsban (0.48) | 3 | + | 1 / m | **5** | 53 | - | 1 / w |
| **6** | 42 | 240 | Daily | Match (0.01); Lorsban (0.48) | 1 | - | 2 / m | **6** | 42 | - | 1 / m |
| **7** | 55 | 12 | Once a week | Lorsban (0.48); Parathion (0.09) | 10 | - | 2 / y | **7** | 55 | - | - |
| **8** | 35 | 216 | Once a week | Carbosulfan (1.2); Mancozeb (2); Furadan (0.72); Lorsban (0.48); Cerillo (0.552); Curacron (0.4) | 1.5 | - | 1 / m | **8** | 35 | - | - |
| **9** | 39 | 39 | Once a week | Paraquat (0.3); Mancozeb (2) | 1 | - | 1 / w | **9** | 38 | - | - |
| **10** | 56 | 528 | Daily | Antracol (2); Cerillo (0.552); Eltra (1.2); Lorsban (0.48); Mancozeb (2); Curacron (0.4); Furadan (0.72); Cymozeb (1.5) | 2 | + | 2 / w | **10** | 55 | - | - |
| **11** | 37 | 120 | Between 2 to 5 months | Tiguvon (2.5 ml x100 kg of bovine weight) | 1 | - | - | **11** | 38 | - | 1 / m |
| **12** | 53 | 360 | Once a week | Cerillo (0.552); Cymozeb (1.5); Fulminator (0.15) | 1 | + | 1 / w | **12** | 51 | - | - |
| **13** | 35 | 180 | Biweekly | Mancozeb (2); Furadan (0.72) | 1.286 | - | - | **13** | 36 | - | 1 / m |
| **14** | 43 | 120 | Monthly | Curacron (0.4); Furadan (0.72); Lorsban (0.48); Lannate (0.45) | 2 | - | 4 / m | **14** | 44 | - | - |
| **15** | 51 | 108 | Each 15 days | Antracol (2); Mancozeb (2); Fitoraz (1.5) | 4 | - | 1 / m | **15** | 49 | - | - |
| **16** | 58 | 360 | Monthly | Mancozeb (2); Perban (1) | 1 | - | - | **16** | 56 | + | 1 / m |
| **17** | 27 | 84 | Biweekly | Antracol (2); Cerillo (0.552); Curacron (0.4); Cymozeb (1.5); Eltra (1.2) | 8 | - | 1 / m | **17** | 28 | - | 1 / m |
| **18** | 44 | 240 | Between 2 to 5 months | Antracol (2); Cerillo (0.552); Fulminator (0.25) | 2 | - | 1 / m | **18** | 45 | + | - |
| **19** | 30 | 120 | Each 15 days | Mancozeb (2); Furadan (0.72); Curacron (0.4) | 1.286 | - | 1 / m | **19** | 29 | + | 4 / m |
| **20** | 54 | 36 | Between 2 to 5 months | Mancozeb (2) | 1 | - | - | **20** | 54 | - | 1 / m |
| **21** | 33 | 144 | Once a week | Carbosulfan (1.2); Cayenne (0.11); Cypermethrin (0.045) | 5 | - | 1 / m | **21** | 32 | - | 1 / m |
| **22** | 23 | 6 | Once a week | Paraquat (0.3); Mancozeb (2) | 1 | - | 4 / m | **22** | 23 | - | - |
| **23** | 58 | 12 | Once a week | Antracol (2); Cerillo (0.552); Forum (0.8) | 2 | - | 1 / w | **23** | 58 | - | - |
| **24** | 49 | 36 | Once a week | Cypermethrin (0.045) | 1 | - | 1 / m | **24** | 50 | - | 1 / m |
| **25** | 62 | 120 | Once a week | Paraquat (0.3); Mancozeb (2) | 1 | - | 1 / m | **25** | 63 | - | 1 / w |
| **26** | 24 | 24 | Between 2 to 5 months | Decis (0.0125) | 1 | - | 1 / m | **26** | 23 | - | - |
| **27** | 47 | 12 | Once a week | Forum (0.8); Dithane (2) | 1 | - | 1 / m | **27** | 45 | - | - |
| **28** | 48 | 60 | Once a week | Furadan (0.72); Lorsban (0.48); Arrivo (0.08) | 2 | - | 4 / m | **28** | 46 | - | - |
| **29** | 55 | 3 | Once a week | Lorsban (0.48); Fulminator (0.1) | 15 | - | - | **29** | 57 | - | - |
| **30** | 35 | 180 | Once a week | Paraquat (0.3); Mancozeb (2) | 1 | - | - | **30** | 34 | - | - |
| **31** | 41 | 168 | Monthly | Lorsban (0.48); Curacron (0.4); Karate (0.015) | 1 - 2 | - | - | **31** | 42 | - | 1 / m |
| **32** | 70 | 24 | Each 15 days | Lorsban (0.48); Curacron (0.4); Decis (0.0125) | 1 | + | 2 / m | **32** | 68 | - | 1 / w |
| **33** | 39 | 120 | Four times a week | Antracol (2); Confidor (0.21) | 2 | - | 1 / w | **33** | 37 | - | 1 / m |
| **34** | 58 | 84 | Once a week | Astuto (0.032); Engeo (0.028) | 1 | - | 1 / w | **34** | 56 | + | - |
| **Mean** | 46,65 | 133,23529 |  |  |  |  | | **Mean** | 46,24 |  | |
| **Median** | 48,5 | 120 |  |  |  |  |  | **Median** | 47,5 |  |  |
| **SD** | 12,13 | 126,7 |  |  |  |  |  | **SD** | 11,92 |  |  |

**Abbreviations:** E, Exposed; UE, unexposed; Kg, Kilograms; Ha, Hectare; S, Smoking; D, Drinking; m, month; w, week; y, year; SD, Standard Deviation

**Supplementary Table 2.** Chromosomal Instability (CIN), Shannon Diversity Index (SDI) and True Diversity Index (TD) for Exposed (E) and Unexposed (UE) groups.

| **E** | **CIN** | | | | | | | **SID** | | | | | | | **TD** | | | | | | |
| --- | --- | --- | --- | --- | --- | --- | --- | --- | --- | --- | --- | --- | --- | --- | --- | --- | --- | --- | --- | --- | --- |
|  | **CEP2** | **CEP3** | **CEP8** | **CEP11** | **CEP15** | **CEP17** | **Mean** | **CEP2** | **CEP3** | **CEP8** | **CEP11** | **CEP15** | **CEP17** | **Mean** | **CEP2** | **CEP3** | **CEP8** | **CEP11** | **CEP15** | **CEP17** | **Mean** |
| 1 | 51 | 50 | 27 | 31 | 57 | 32 | 41,333 | 1,503 | 1,4052 | 0,7378 | 0,8935 | 1,3923 | 0,8895 | 1,1369 | 4,4952 | 4,0763 | 2,0913 | 2,4436 | 4,0243 | 2,434 | 3,2608 |
| 2 | 48 | 44 | 30 | 37 | 46 | 40 | 40,833 | 1,2797 | 1,2543 | 0,9335 | 0,9862 | 1,1254 | 0,9618 | 1,0902 | 3,5957 | 3,5055 | 2,5435 | 2,681 | 3,0814 | 2,6165 | 3,0039 |
| 3 | 46 | 40 | 40 | 44 | 44 | 26 | 40 | 1,3074 | 1,219 | 1,0471 | 1,2124 | 1,1823 | 0,8102 | 1,1297 | 3,6965 | 3,3838 | 2,8494 | 3,3615 | 3,2618 | 2,2483 | 3,1336 |
| 4 | 60 | 47 | 43 | 38 | 44 | 39 | 45,167 | 1,4531 | 1,2609 | 1,1282 | 1,0883 | 1,1437 | 1,2098 | 1,214 | 4,2766 | 3,5286 | 3,0901 | 2,9693 | 3,1385 | 3,3527 | 3,3926 |
| 5 | 62 | 41 | 46 | 32 | 36 | 44 | 43,5 | 1,5442 | 1,3146 | 1,4081 | 1,0722 | 1,15 | 1,2676 | 1,2928 | 4,6841 | 3,7234 | 4,0881 | 2,9217 | 3,1583 | 3,5524 | 3,688 |
| 6 | 40 | 69 | 69 | 56 | 66 | 69 | 61,5 | 1,1504 | 1,5432 | 1,6958 | 1,101 | 1,4692 | 1,6732 | 1,4388 | 3,1596 | 4,6794 | 5,4509 | 3,0072 | 4,3456 | 5,3293 | 4,3286 |
| 7 | 33 | 56 | 26 | 51 | 56 | 36 | 43 | 0,7561 | 1,1971 | 0,7637 | 0,8777 | 1,142 | 0,93 | 0,9444 | 2,1299 | 3,3106 | 2,1462 | 2,4053 | 3,1331 | 2,5345 | 2,6099 |
| 8 | 42 | 48 | 30 | 43 | 37 | 30 | 38,333 | 1,0673 | 1,1851 | 0,8343 | 0,9619 | 0,9546 | 0,8602 | 0,9772 | 2,9075 | 3,2711 | 2,3033 | 2,6167 | 2,5977 | 2,3636 | 2,6767 |
| 9 | 34 | 44 | 22 | 41 | 48 | 22 | 35,167 | 0,8761 | 0,9682 | 0,6448 | 0,9064 | 0,8675 | 0,5013 | 0,7941 | 2,4016 | 2,6333 | 1,9056 | 2,4754 | 2,3809 | 1,6509 | 2,2413 |
| 10 | 40 | 29 | 23 | 38 | 42 | 15 | 31,167 | 0,9838 | 0,8132 | 0,6597 | 0,9046 | 0,8124 | 0,5182 | 0,782 | 2,6746 | 2,255 | 1,9342 | 2,471 | 2,2533 | 1,679 | 2,2112 |
| 11 | 31 | 41 | 18 | 52 | 45 | 17 | 34 | 0,9089 | 0,9741 | 0,5525 | 0,9607 | 0,8468 | 0,6051 | 0,808 | 2,4815 | 2,6488 | 1,7376 | 2,6134 | 2,3321 | 1,8315 | 2,2742 |
| 12 | 30 | 32 | 23 | 33 | 45 | 26 | 31,5 | 0,761 | 0,8849 | 0,6597 | 0,679 | 0,7984 | 0,6847 | 0,7446 | 2,1404 | 2,4228 | 1,9342 | 1,9719 | 2,2219 | 1,9831 | 2,1124 |
| 13 | 34 | 23 | 21 | 39 | 29 | 15 | 26,833 | 0,8375 | 0,6072 | 0,6292 | 0,8794 | 0,6986 | 0,5237 | 0,6959 | 2,3107 | 1,8353 | 1,8761 | 2,4095 | 2,0109 | 1,6882 | 2,0218 |
| 14 | 28 | 22 | 25 | 37 | 31 | 16 | 26,5 | 0,665 | 0,5939 | 0,6541 | 0,8481 | 0,7811 | 0,5493 | 0,6819 | 1,9445 | 1,8111 | 1,9233 | 2,3352 | 2,1838 | 1,7321 | 1,9883 |
| 15 | 29 | 30 | 29 | 43 | 27 | 39 | 32,833 | 0,8043 | 0,8802 | 0,6986 | 0,8899 | 0,8181 | 0,626 | 0,7862 | 2,2351 | 2,4115 | 2,0109 | 2,4349 | 2,2662 | 1,8702 | 2,2048 |
| 16 | 34 | 30 | 32 | 39 | 39 | 16 | 31,667 | 1,0161 | 0,948 | 0,7017 | 1,019 | 0,8254 | 0,5455 | 0,8426 | 2,7625 | 2,5805 | 2,0171 | 2,7705 | 2,2829 | 1,7255 | 2,3565 |
| 17 | 26 | 44 | 21 | 38 | 49 | 18 | 32,667 | 0,7245 | 0,82 | 0,5542 | 0,837 | 0,8315 | 0,5667 | 0,7223 | 2,0637 | 2,2704 | 1,7405 | 2,3095 | 2,2967 | 1,7625 | 2,0739 |
| 18 | 41 | 22 | 32 | 55 | 33 | 23 | 34,333 | 0,9046 | 0,6932 | 0,795 | 1,0591 | 0,817 | 0,7246 | 0,8323 | 2,471 | 2,0002 | 2,2144 | 2,8839 | 2,2636 | 2,0639 | 2,3162 |
| 19 | 30 | 44 | 21 | 37 | 48 | 28 | 34,667 | 0,7941 | 0,9089 | 0,6574 | 0,8956 | 0,9388 | 0,7754 | 0,8284 | 2,2125 | 2,4815 | 1,9297 | 2,4489 | 2,5568 | 2,1716 | 2,3001 |
| 20 | 33 | 31 | 18 | 37 | 37 | 19 | 29,167 | 0,8505 | 0,814 | 0,595 | 0,9404 | 0,8384 | 0,6491 | 0,7812 | 2,3408 | 2,257 | 1,8131 | 2,561 | 2,3127 | 1,9137 | 2,1997 |
| 21 | 38 | 35 | 19 | 43 | 34 | 26 | 32,5 | 0,9047 | 0,9232 | 0,5857 | 1,0483 | 0,8672 | 0,7408 | 0,845 | 2,4713 | 2,5173 | 1,7962 | 2,8527 | 2,3802 | 2,0975 | 2,3526 |
| 22 | 44 | 32 | 27 | 46 | 36 | 18 | 33,833 | 0,9611 | 0,8386 | 0,7865 | 0,9173 | 0,8441 | 0,5962 | 0,824 | 2,6147 | 2,3131 | 2,1957 | 2,5026 | 2,3259 | 1,8151 | 2,2945 |
| 23 | 37 | 33 | 26 | 36 | 31 | 38 | 33,5 | 0,8055 | 0,8951 | 0,7245 | 0,8559 | 0,814 | 0,8596 | 0,8258 | 2,2378 | 2,4477 | 2,0637 | 2,3534 | 2,257 | 2,3623 | 2,287 |
| 24 | 44 | 37 | 38 | 45 | 30 | 33 | 37,833 | 0,9743 | 0,8749 | 0,8927 | 0,8648 | 0,8732 | 0,8047 | 0,8808 | 2,6494 | 2,3985 | 2,4417 | 2,3746 | 2,3945 | 2,236 | 2,4158 |
| 25 | 31 | 32 | 24 | 34 | 42 | 25 | 31,333 | 0,7714 | 0,945 | 0,6199 | 0,8139 | 0,8336 | 0,6541 | 0,773 | 2,1628 | 2,5728 | 1,8588 | 2,2567 | 2,3016 | 1,9233 | 2,1793 |
| 26 | 41 | 40 | 27 | 39 | 48 | 38 | 38,833 | 0,955 | 0,9498 | 0,7702 | 0,9328 | 0,8917 | 0,8721 | 0,8953 | 2,5988 | 2,5851 | 2,1602 | 2,5415 | 2,4394 | 2,3919 | 2,4528 |
| 27 | 47 | 38 | 30 | 57 | 26 | 44 | 40,333 | 1,0428 | 0,7848 | 0,7848 | 0,9805 | 0,82 | 0,5731 | 0,831 | 2,837 | 2,1921 | 2,1921 | 2,6659 | 2,2704 | 1,7737 | 2,3219 |
| 28 | 47 | 40 | 24 | 51 | 42 | 21 | 37,5 | 0,9471 | 0,7999 | 0,6199 | 1,0476 | 0,8695 | 0,6292 | 0,8189 | 2,5782 | 2,2253 | 1,8588 | 2,8508 | 2,3858 | 1,8761 | 2,2958 |
| 29 | 37 | 32 | 20 | 42 | 48 | 28 | 34,5 | 0,8841 | 0,8068 | 0,5849 | 0,7607 | 0,83 | 0,665 | 0,7553 | 2,4209 | 2,2408 | 1,7949 | 2,1398 | 2,2934 | 1,9445 | 2,139 |
| 30 | 33 | 41 | 11 | 41 | 32 | 15 | 28,833 | 0,817 | 0,9488 | 0,3465 | 0,8475 | 0,6714 | 0,4594 | 0,6818 | 2,2636 | 2,5826 | 1,4141 | 2,3339 | 1,9569 | 1,5832 | 2,0224 |
| 31 | 36 | 73 | 15 | 34 | 33 | 14 | 34,167 | 0,93 | 0,7865 | 0,4816 | 0,7642 | 0,7347 | 0,441 | 0,6897 | 2,5345 | 2,1957 | 1,6187 | 2,1472 | 2,0849 | 1,5542 | 2,0226 |
| 32 | 32 | 22 | 10 | 33 | 28 | 11 | 22,667 | 0,873 | 0,6558 | 0,3576 | 0,679 | 0,593 | 0,3987 | 0,5928 | 2,3941 | 1,9267 | 1,4299 | 1,9719 | 1,8093 | 1,4898 | 1,837 |
| 33 | 30 | 39 | 8 | 23 | 30 | 13 | 23,833 | 0,8555 | 1,0167 | 0,3089 | 0,5804 | 0,7848 | 0,4216 | 0,6613 | 2,3527 | 2,7642 | 1,3619 | 1,7868 | 2,1921 | 1,5245 | 1,997 |
| 34 | 30 | 40 | 17 | 28 | 21 | 19 | 25,833 | 0,8018 | 0,9422 | 0,5175 | 0,6883 | 0,58 | 0,5254 | 0,6759 | 2,2296 | 2,5657 | 1,6778 | 1,9903 | 1,786 | 1,6911 | 1,9901 |
| **UE** | **CIN** | | | | | | | **SID** | | | | | | | **TD** | | | | | | |
|  | **CEP2** | **CEP3** | **CEP8** | **CEP11** | **CEP15** | **CEP17** | **Mean** | **CEP2** | **CEP3** | **CEP8** | **CEP11** | **CEP15** | **CEP17** | **Mean** | **CEP2** | **CEP3** | **CEP8** | **CEP11** | **CEP15** | **CEP17** | **Mean** |
| 1 | 18 | 18 | 10 | 10 | 16 | 11 | 13,833 | 0,7054 | 0,638 | 0,4583 | 0,4053 | 0,651 | 0,4463 | 0,5507 | 2,0246 | 1,8927 | 1,5814 | 1,4997 | 1,9175 | 1,5626 | 1,7464 |
| 2 | 13 | 7 | 11 | 12 | 10 | 2 | 9,1667 | 0,498 | 0,2955 | 0,456 | 0,4658 | 0,3862 | 0,1119 | 0,3689 | 1,6455 | 1,3438 | 1,5777 | 1,5933 | 1,4713 | 1,1184 | 1,4583 |
| 3 | 6 | 1 | 2 | 3 | 8 | 2 | 3,6667 | 0,2652 | 0,056 | 0,098 | 0,1538 | 0,3317 | 0,098 | 0,1671 | 1,3036 | 1,0576 | 1,103 | 1,1663 | 1,3933 | 1,103 | 1,1878 |
| 4 | 13 | 12 | 2 | 6 | 4 | 10 | 7,8333 | 0,5067 | 0,471 | 0,1119 | 0,2877 | 0,1904 | 0,3862 | 0,3256 | 1,6597 | 1,6016 | 1,1184 | 1,3333 | 1,2098 | 1,4713 | 1,399 |
| 5 | 15 | 8 | 1 | 4 | 9 | 9 | 7,6667 | 0,5896 | 0,3844 | 0,056 | 0,1904 | 0,3869 | 0,4104 | 0,3363 | 1,8032 | 1,4688 | 1,0576 | 1,2098 | 1,4723 | 1,5074 | 1,4199 |
| 6 | 15 | 4 | 10 | 14 | 13 | 9 | 10,833 | 0,4227 | 0,1904 | 0,3576 | 0,441 | 0,4422 | 0,3025 | 0,3594 | 1,5261 | 1,2098 | 1,4299 | 1,5542 | 1,5561 | 1,3533 | 1,4382 |
| 7 | 11 | 6 | 7 | 9 | 7 | 5 | 7,5 | 0,3987 | 0,2652 | 0,2955 | 0,3025 | 0,2536 | 0,1985 | 0,2857 | 1,4898 | 1,3036 | 1,3438 | 1,3533 | 1,2887 | 1,2196 | 1,3331 |
| 8 | 12 | 5 | 9 | 13 | 5 | 1 | 7,5 | 0,3465 | 0,2322 | 0,3502 | 0,4216 | 0,2235 | 0,056 | 0,2717 | 1,4141 | 1,2613 | 1,4194 | 1,5245 | 1,2505 | 1,0576 | 1,3212 |
| 9 | 5 | 1 | 0 | 2 | 4 | 2 | 2,3333 | 0,246 | 0,056 | 0 | 0,098 | 0,1679 | 0,098 | 0,111 | 1,2789 | 1,0576 | 1 | 1,103 | 1,1829 | 1,103 | 1,1209 |
| 10 | 8 | 6 | 6 | 16 | 7 | 5 | 8 | 0,3342 | 0,254 | 0,227 | 0,4397 | 0,2823 | 0,1985 | 0,2893 | 1,3969 | 1,2892 | 1,2548 | 1,5522 | 1,3262 | 1,2196 | 1,3398 |
| 11 | 8 | 9 | 2 | 6 | 8 | 6 | 6,5 | 0,3089 | 0,3339 | 0,098 | 0,227 | 0,2788 | 0,227 | 0,2456 | 1,3619 | 1,3964 | 1,103 | 1,2548 | 1,3215 | 1,2548 | 1,2821 |
| 12 | 11 | 6 | 6 | 8 | 8 | 6 | 7,5 | 0,3465 | 0,227 | 0,254 | 0,3089 | 0,3089 | 0,227 | 0,2787 | 1,4141 | 1,2548 | 1,2892 | 1,3619 | 1,3619 | 1,2548 | 1,3228 |
| 13 | 10 | 6 | 5 | 8 | 4 | 4 | 6,1667 | 0,3251 | 0,254 | 0,1985 | 0,2788 | 0,1679 | 0,1679 | 0,232 | 1,3841 | 1,2892 | 1,2196 | 1,3215 | 1,1829 | 1,1829 | 1,2634 |
| 14 | 13 | 12 | 7 | 9 | 13 | 9 | 10,5 | 0,3864 | 0,3669 | 0,2536 | 0,3025 | 0,3025 | 0,3251 | 0,3229 | 1,4717 | 1,4433 | 1,2887 | 1,3533 | 1,3533 | 1,3841 | 1,3824 |
| 15 | 15 | 4 | 10 | 12 | 15 | 4 | 10 | 0,5263 | 0,3007 | 0,3251 | 0,3669 | 0,3669 | 0,1904 | 0,3461 | 1,6927 | 1,3508 | 1,3841 | 1,4433 | 1,4433 | 1,2098 | 1,4207 |
| 16 | 6 | 9 | 1 | 4 | 9 | 7 | 6 | 0,2652 | 0,3598 | 0,056 | 0,1679 | 0,2546 | 0,2536 | 0,2262 | 1,3036 | 1,4331 | 1,0576 | 1,1829 | 1,29 | 1,2887 | 1,2593 |
| 17 | 10 | 8 | 7 | 12 | 6 | 2 | 7,5 | 0,3751 | 0,3025 | 0,2823 | 0,4013 | 0,227 | 0,2536 | 0,307 | 1,4552 | 1,3533 | 1,3262 | 1,4938 | 1,2548 | 1,2887 | 1,362 |
| 18 | 13 | 8 | 7 | 16 | 12 | 8 | 10,667 | 0,4216 | 0,2788 | 0,2536 | 0,4397 | 0,3669 | 0,1347 | 0,3159 | 1,5245 | 1,3215 | 1,2887 | 1,5522 | 1,4433 | 1,1442 | 1,3791 |
| 19 | 12 | 9 | 5 | 13 | 11 | 4 | 9 | 0,3669 | 0,3025 | 0,1985 | 0,3864 | 0,3987 | 0,1904 | 0,3072 | 1,4433 | 1,3533 | 1,2196 | 1,4717 | 1,4898 | 1,2098 | 1,3646 |
| 20 | 4 | 8 | 3 | 7 | 8 | 4 | 5,6667 | 0,1957 | 0,3317 | 0,1538 | 0,2955 | 0,3089 | 0,1679 | 0,2423 | 1,2161 | 1,3933 | 1,1663 | 1,3438 | 1,3619 | 1,1829 | 1,2774 |
| 21 | 9 | 5 | 8 | 3 | 5 | 11 | 6,8333 | 0,3644 | 0,2235 | 0,2788 | 0,1347 | 0,2235 | 0,3465 | 0,2619 | 1,4396 | 1,2505 | 1,3215 | 1,1442 | 1,2505 | 1,4141 | 1,3034 |
| 22 | 8 | 6 | 5 | 8 | 5 | 6 | 6,3333 | 0,3317 | 0,254 | 0,2235 | 0,3089 | 0,1985 | 0,227 | 0,2573 | 1,3933 | 1,2892 | 1,2505 | 1,3619 | 1,2196 | 1,2548 | 1,2949 |
| 23 | 3 | 6 | 2 | 2 | 15 | 5 | 5,5 | 0,1538 | 0,2686 | 0,098 | 0,098 | 0,4227 | 0,1985 | 0,2066 | 1,1663 | 1,3081 | 1,103 | 1,103 | 1,5261 | 1,2196 | 1,2377 |
| 24 | 6 | 7 | 4 | 5 | 6 | 3 | 5,1667 | 0,254 | 0,2823 | 0,1904 | 0,1985 | 0,1538 | 0,1538 | 0,2055 | 1,2892 | 1,3262 | 1,2098 | 1,2196 | 1,1663 | 1,1663 | 1,2296 |
| 25 | 5 | 5 | 3 | 3 | 8 | 2 | 4,3333 | 0,2235 | 0,2235 | 0,1347 | 0,1347 | 0,3089 | 0,098 | 0,1873 | 1,2505 | 1,2505 | 1,1442 | 1,1442 | 1,3619 | 1,103 | 1,2091 |
| 26 | 6 | 4 | 1 | 6 | 8 | 2 | 4,5 | 0,2652 | 0,1679 | 0,056 | 0,254 | 0,2788 | 0,098 | 0,1867 | 1,3036 | 1,1829 | 1,0576 | 1,2892 | 1,3215 | 1,103 | 1,2096 |
| 27 | 12 | 9 | 7 | 20 | 8 | 6 | 10,333 | 0,4484 | 0,3339 | 0,2536 | 0,5004 | 0,2788 | 0,227 | 0,3404 | 1,5658 | 1,3964 | 1,2887 | 1,6494 | 1,3215 | 1,2548 | 1,4128 |
| 28 | 4 | 4 | 3 | 9 | 11 | 5 | 6 | 0,1679 | 0,1957 | 0,1538 | 0,3025 | 0,3465 | 0,1985 | 0,2275 | 1,1829 | 1,2161 | 1,1663 | 1,3533 | 1,4141 | 1,2196 | 1,2587 |
| 29 | 8 | 3 | 6 | 5 | 3 | 1 | 4,3333 | 0,3342 | 0,3339 | 0,227 | 0,1985 | 0,1347 | 0,056 | 0,2141 | 1,3969 | 1,3964 | 1,2548 | 1,2196 | 1,1442 | 1,0576 | 1,2449 |
| 30 | 3 | 1 | 0 | 0 | 0 | 1 | 0,8333 | 0,1078 | 0,056 | 0 | 0 | 0 | 0,056 | 0,0366 | 1,1138 | 1,0576 | 1 | 1 | 1 | 1,0576 | 1,0382 |
| 31 | 3 | 93 | 0 | 2 | 2 | 1 | 16,833 | 0,1347 | 0,2536 | 0 | 0,098 | 0,098 | 0,056 | 0,1067 | 1,1442 | 1,2887 | 1 | 1,103 | 1,103 | 1,0576 | 1,1161 |
| 32 | 4 | 1 | 0 | 3 | 1 | 0 | 1,5 | 0,1679 | 0,056 | 0 | 0,1347 | 0,056 | 0 | 0,0691 | 1,1829 | 1,0576 | 1 | 1,1442 | 1,0576 | 1 | 1,0737 |
| 33 | 2 | 5 | 0 | 2 | 2 | 1 | 2 | 0,098 | 0,1985 | 0 | 0,098 | 0,098 | 0,056 | 0,0914 | 1,103 | 1,2196 | 1 | 1,103 | 1,103 | 1,0576 | 1,0977 |
| 34 | 3 | 2 | 0 | 1 | 3 | 1 | 1,6667 | 0,1347 | 0,098 | 0 | 0,056 | 0,1347 | 0,056 | 0,0799 | 1,1442 | 1,103 | 1 | 1,0576 | 1,1442 | 1,0576 | 1,0844 |

**Abbreviations:** E, Exposed individuals; UE, Unexposed individuals; CEP2, centromeric probe for chromosome 2; CEP3, centromeric probe for chromosome 3; CEP8, centromeric probe for chromosome 8; CEP11, centromeric probe for chromosome 11; CEP15, centromeric probe for chromosome 15; CEP17, centromeric probe for chromosome 17; CIN, Chromosomal Instability; SDI, Shannon Diversity Index; TD, True Diversity index

**Supplementary Figure 1**. Clonal heterogeneity (CH) determined by Shannon Diversity Index (SDI) for exposed and unexposed groups. Values below 1.5 were considered indicative of low CH, values between 1.6 and 2 were considered indicative of intermediate CH; and values higher than 2 were considered indicative of high CH.

**Supplementary Figure 2**. Clonal heterogeneity (CH) determined by True Diversity index (TD) for each chromosome evaluated in the (A) Exposed and (B) Unexposed groups. According to the level of CIN, each chromosome was classified as having low CIN (CIN<25%) or high CIN (CIN≥25%). The CH for each chromosome was classified as low CH (CH<1.5), intermediate CH (CH>1.6<2) or high (CH>2). The chromosome with the lowest CH for the exposed group was chromosome 17, and the one with the highest CH was chromosome 3. Likewise, for unexposed group, the chromosome with the lowest CH was chromosome 17 and the chromosome with the highest CH was chromosome 2.

**Supplementary Figure 3**. Clonal heterogeneity (CH) determined by Shannon Diversity Index (SDI) for each chromosome evaluated in the (A) Exposed and (B) Unexposed groups. According to the level of CIN, each chromosome was classified as having low CIN (CIN<25%) or high CIN (CIN≥25%). The CH for each chromosome was classified as low CH (CH<1.5), intermediate CH (CH>1,6<2) or high (CH>2). The chromosome with the lowest CH for the exposed group was chromosome 17, and the one with the highest CH was chromosome 3. Likewise, for unexposed group, the chromosome with the lowest CH was chromosome 17 and the chromosome with the highest CH was chromosome 2.
